# Optimizing superparamagnetic ferrite nanoparticles: microwave-assisted *vs.* thermal decomposition synthesis methods†

**DOI:** 10.1039/d5na00244c

**Published:** 2025-05-26

**Authors:** Kimia Moghaddari, Lars Schumacher, Rainer Pöttgen, Guido Kickelbick

**Affiliations:** a Inorganic Solid-State Chemistry, Saarland University Campus, Building C4 1 66123 Saarbrücken Germany guido.kickelbick@uni-saarland.de +49-681-302-70651; b Institut für Anorganische und Analytische Chemie, Universität Münster Corrensstrasse 30 48149 Münster Germany; c Saarene – Saarland Center for Energy Materials and Sustainability Campus C4 2 66123 Saarbrücken Germany

## Abstract

Superparamagnetic iron oxide nanoparticles are of crucial importance for various applications in medicine and biology as well as in materials science, where properties such as magnetism and inductive heating are advantageous. In this study, we systematically compare the synthesis methods for ferrite nanoparticles with those of pure iron oxide, focusing on their final properties. We synthesized superparamagnetic substituted ferrite nanoparticles with an average diameter of 5 to 8 nm with the general formula of M_*x*_Fe_3−*x*_O_4_ (M = Fe^2+^, Mn^2+^, Co^2+^) using both conventional thermal decomposition (TD) method and microwave-assisted (MW) methods. Although the manganese-substituted particles obtained through both methods exhibited a narrow size distribution and high surface coverage with oleic acid, they demonstrated lower heating efficiency in an induction field compared to the cobalt-substituted particles. In particular, the replacement of Fe^2+^ ions with Co^2+^ ions significantly improved the self-heating ability and increased the specific absorption rate (SAR) from 22.7 for Fe_3_O_4_ to 106.3 W g_NP_^−1^ for Co_0.88_Fe_2.12_O_4_ nanoparticles. In addition, the concentration of 1,2-dodecanediol in the reaction mixture significantly influenced the shape and size distribution of the particles. Microwave-assisted synthesis resulted in higher incorporation of M^2+^ ions, as confirmed by ICP-MS and EDX spectroscopy, and more uniform particle sizes due to homogeneous nucleation. By optimizing the microwave method, we were able to produce small size superparamagnetic particles with high saturation magnetization (89.2 emu g^−1^ at 300 K), capable of generating more heat in the magnetic field, making these particles suitable candidates for induction heating in materials.

## Abstract 2

Superparamagnetic ferrite nanoparticles with high heating power and magnetization were synthesized using an environmentally friendly, microwave-assisted method. The particles are excellent candidates for induction heating in materials.

## Graphical abstract

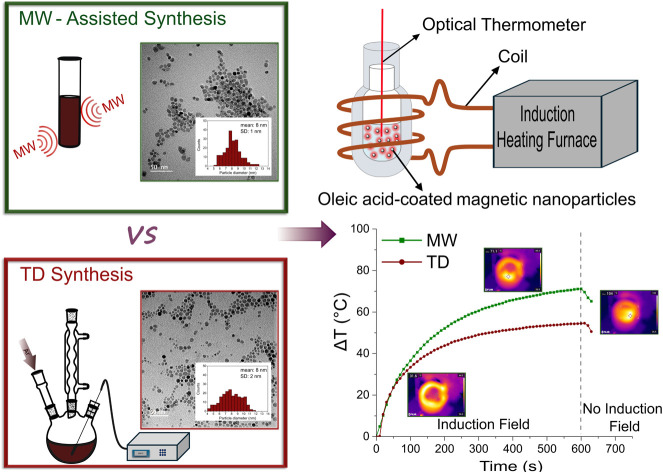

## Introduction

In recent years, the synthesis of magnetic nanoparticles has attracted considerable attention due to their unique physical and chemical properties and their wide range of applications. Beyond their biomedical applications, particularly as MRI contrast agents,^1^ in drug delivery systems,^2^ and in hyperthermia-based cancer treatments,^3^ these nanoparticles play a crucial role in advancing smart materials. They have been explored in the field of material science for the preparation of sensors,^4^ batteries,^5^ and, notably, in self-healing materials^6,7^ and debonding-on-demand systems.^8^ The most widely used types of magnetic nanoparticles are iron oxide nanoparticles (IONPs) mostly known in the form of hematite (α-Fe_2_O_3_), maghemite (γ-Fe_2_O_3_), and magnetite (Fe_3_O_4_).^9^ Among the IONPs, magnetite (Fe_3_O_4_) is used more frequently due to its higher saturation magnetization. The structure of magnetite is described as an inverse spinel structure in which oxygen anions form a cubic closed packed substructure, in which Fe^2+^ cations occupy ¼ of the octahedral sites and Fe^3+^ cations occupy ¼ of the octahedral and ⅛ of the tetrahedral sites.^10,11^ Bulk magnetite exhibits a ferrimagnetic behavior. Reducing the particle size to less than 20 nm leads to the formation of a single magnetic domain and the generation of a superparamagnetic material, *i.e.*, the nanoparticles show magnetism only in the presence of a magnetic field.^12,13^ In the presence of an alternating magnetic field, superparamagnetic nanoparticles generate heat through Néel and Brownian relaxation. In Néel relaxation, the external field causes magnetic moments to reorient, releasing stored magnetic energy in the form of heat. In Brownian relaxation, the particles physically rotate, generating heat through shear interactions with the surrounding medium.^14,15^ Thus, the unique properties of superparamagnetic nanoparticles make them particularly valuable for heat generation in materials through magnetic induction, offering precise control at high temperatures, especially for heating the interior regions of a material. For self-healing inorganic–organic nanocomposites^16–18^ or debonding on demand systems,^19,20^ superparamagnetic nanoparticles with higher heating efficiencies are preferred. Metal ferrite MFe_2_O_4_ (M = Mn^2+^, Co^2+^, Ni^2+^, Mg^2+^, Zn^2+^, *etc.*) nanoparticles seem to be good candidates due to their higher saturation magnetization, larger Curie temperature and effective anisotropy in comparison to iron oxide nanoparticles.^21,22^ The magnetic properties of the nanoparticles can be largely influenced by parameters such as size and size distribution, morphology, chemical composition and surface functionalization, which can be adjusted by choosing a proper synthesis method (Table 1).^23^ Chemical synthesis routes are preferred over physical methods as they allow better control over the size of the particles.^24,25^ Among the chemical synthesis methods, microemulsion,^26,27^ co-precipitation,^28,29^ thermal decomposition^30–32^ and recently, microwave-assisted methods^12,33^ are the most widely used routes. The decomposition of metal-containing precursors in an organic solvent is a highly effective method for producing small size monodisperse nanoparticles compared to precipitation with salts, which often results in extensive agglomeration of the particles and very broad size distributions. In addition, the thermal decomposition method offers the possibility to control and modify the surface properties of the particles by using different agents for surface coating, resulting in particles with high dispersibility in matrices with different polarities, which is beneficial for further applications in materials science.^25,32,34^ On the other hand, microwave-assisted method offers an attractive alternative to conventional methods while retaining all the advantages of the thermal decomposition method. In this approach, the heat will be generated directly within the reaction mixture resulting in homogeneous nucleation. In addition, this method has the advantage of a high reproducibility, faster reaction time, and low energy costs, which makes it an environmentally friendly method.^24^

**Table 1:** Comparison of SAR value determinations of various ferrite nanoparticles regarding effective parameters on magnetic properties

Magnetic particles	Synthesis method^a^^,^^b^^,^^c^	*D*_TEM_ d (nm)	Surface coverage	Field strength	*ν* (kHz)	SAR (W g^−1^)	Ref.
Fe_3_O_4_	TD	8	OA	47.7 kA m^−1^	194	30.1^e^	35
Fe_3_O_4_	TD	9	mPEG (2000 Da)	27 kA m^−1^	400	367^e^	36
Fe_3_O_4_	CP	10	Polyacrylic acid	15 mT	308	36.5–37.3^f^	37
Fe_3_O_4_	CP	8	—	23.51 kA m^−1^	312	39.50^e^	38
Fe_3_O_4_	CP	10	OA	23.51 kA m^−1^	312	45.98^e^	38
Fe_3_O_4_	MW-TD	6	OA	12 A	390	158^f^	39
Fe_2_O_3_	TD	16	—	38.2 kA m^−1^	430	249.1^e^	35
MnFe_2_O_4_	CP	25	—	4 kA m^−1^	280	217.62^f^	40
MnFe_2_O_4_	CP	19	—	3 mT	1950	68.7^f^	41
Co_0.1_Fe_2.9_O_4_	CP	13.5	—	300 Oe	450	296.8^f^	42
Co_0.5_Fe_2.5_O_4_	CP	20.8	—	300 Oe	450	183.9^f^	42
CoFe_2_O_4_	CP	19.4	—	300 Oe	450	196.5^f^	42
Ni_0.31_Fe_2.69_O_4_	TD	8	OA/OAm	20 kA m^−1^	872	84^e^	43
Ni_0.86_Fe_2.14_O_4_	TD	11.4	OA/OAm	20 kA m^−1^	872	104^e^	43

a Thermal decomposition (TD).

b Co-precipitation (CP).

c MW-assisted thermal decomposition method (MW-TD).

d Diameter of uncoated nanoparticles measured by TEM.

e SAR values were reported as W g_Fe_^−1^ or W g_ferrite_^−1^.

f SAR values were reported as W g_NP_^−1^.

The synthesis of ferrite nanoparticles has traditionally been limited to the production of maghemite and magnetite nanoparticles through microwave-assisted hydrothermal approaches, which often yield particles with moderate heating efficiency. However, to the best of our knowledge, the use of a microwave-assisted system for synthesizing surface-functionalized substituted ferrites, coupled with the thermal decomposition approach, has yet to be explored. This study addresses this gap by employing both thermal decomposition and microwave-assisted methods to synthesize superparamagnetic ferrite nanoparticles. Through a systematic comparison of these methods, we investigate their ability to control particle size, monodispersity, and stoichiometric composition. Moreover, we evaluate the heating efficiency of the resulting ferrites in an alternating magnetic field, focusing on their self-heating capacity derived from superparamagnetic properties. By optimizing the microwave-assisted method, we successfully produce small-sized ferrite nanoparticles with high magnetization and heating efficiency, offering a cost-effective and straightforward alternative to the thermal decomposition method. This approach results in reproducible, highly uniform superparamagnetic particles, making it ideal for nanocomposite inductive heating. Their superparamagnetic nature ensures precise temperature control, enabling us to achieve high temperatures with only a small amount of particles in the materials without compromising crucial properties like mechanical strength or heating the undesired parts of the material.

## Materials and methods

### Materials

Fe(acac)_3_ (≥99%), Co(acac)_2_ (≥99%), Mn(acac)_2_, oleic acid (OA, 98.5%), oleyl amine (OAm, >99%), 1,2-dodecandiol (90%) and benzyl ether (98%) were purchased from Sigma-Aldrich (St. Louis, USA). HCl (Suprapur 30%) purchased from Merck Millipore (Burlington, USA) and HNO_3_ (Rotipuran Supra 69%) provided from Carl Roth (Karlsruhe, Germany). Toluene (99.8%) purchased from Stockmeier (Bielefeld, Germany). Poly(dimethylsiloxane), hydrid terminated (viscosity 2–3 and 100 cSt) obtained from abcr GmbH (Karlsruhe, Germany). Ethanol (99%, denatured with 1% PE) and *n*-hexane were provided from BCD Chemie GmbH (Hamburg, Germany). All chemicals were used without any further purification.

### Characterization

Powder X-ray diffraction (PXRD) patterns were recorded using a Bruker D8-A25-Advance diffractometer (Bruker AXS, Karlsruhe, Germany) with Cu-K_α_ radiation (40 kV, 40 mA, *λ* = 154.0596 pm) and a 12 μm Ni foil to reduce K_β_ radiation. A LYNXEYE 1D detector was used on the secondary beam side. The fluorescence induced background was reduced by detector discrimination. Samples were measured on a Si low background sample holder. Data was recorded in a 2*θ* range from 7 to 120° with a step size of 0.013° and in a total 2 h of scan time. The interpretation of the data and Rietveld refinement was performed on TOPAS 5.^44^

Dynamic light scattering (DLS) measurements were performed using an ALV/CGS-3 compact goniometer system (ALV GmbH, Langen, Germany) with an ALV/LSE-5003 correlator at a 90° measurement angle and a wavelength of 632.8 nm. For each measurement, particles from ethanolic dispersion were magnetically decanted off and redispersed in hexane. Samples were measured after 5 min to ensure reaching equilibrium. Each measurement was performed for 5 runs with the duration of 10 s and results were reported in number weighted form.

Transmission electron microscopy (TEM) was carried out using a JEOL JEM-2010 electron microscope (JEOL, Akishima, Japan). For each measurement, 30 μL of particle dispersion in hexane was applied dropwise either on a Plano S160-3 copper mesh coated with a carbon film or a Plano S147-4 copper mesh coated with a carbon hole film and hexane was evaporated under normal conditions. The ImageJ^45^ software was used for evaluation of average diameter and size distribution of the nanoparticles. The standard deviations of the histograms were calculated using the following formula:1
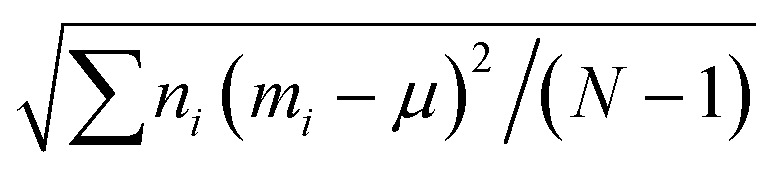
Here, *n*_*i*_ and *m*_*i*_ are the frequency and midpoint of the *i*^th^ bin of the histogram, *μ* is the mean and *N* is the total number of particles determined for each histogram.

Fourier transform infrared (FTIR) spectra were recorded from 4500 to 400 cm^−1^ on attenuated total reflectance (ATR) mode using a Bruker Vertex 70 spectrometer (Bruker Optics, Ettlingen, Germany). Spectra were obtained as an average of 16 scans with a resolution of 4 cm^−1^ and were normalized in correlation with their highest intensity.

Thermogravimetric analyses (TGA) were carried out using a Netzsch TG 209 F1 Iris (Netzsch GmbH, Selb, Germany). About 2–3 mg of the samples were measured in aluminum oxide crucibles with a heating rate of 10 K min^−1^ from room temperature to 900 °C under nitrogen atmosphere followed by heating to 1000 °C under a mixture of nitrogen and oxygen (4 : 1), simulating synthetic air atmosphere.

Scanning electron microscopy-energy dispersive X-ray spectroscopy (SEM-EDX) analyses were carried out using a JEOL 7000F scanning electron microscope (JEOL, Akishima, Japan) coupled with an EDAX Genesis 2000 EDX detector (EDAX, Pleasanton, CA , USA).

Elemental analysis (EA) was performed using an Elementar Vario Micro Cube (Elementar Analysensysteme GmbH, Langenselbold, Germany).

The chemical compositions of the samples were determined by inductively coupled plasma mass spectrometry (ICP-MS). Vacuum-dried particles were dissolved in 4 mL of *aqua regia* (1 : 3 v/v mixture of HNO_3_ and HCl) followed by a dilution with ultra-pure water. The samples were then placed in a shaker to ensure complete dissolution. The measurements were carried out using a commercial ICP-MS system (8900 Triple Quad and SPS4 autosampler, Agilent, Santa Clara, USA). For measurements, stock solutions of single element ICP-MS standards of Fe (Merck Certipur, Darmstadt, Germany), Co (Fluka, Buchs, Switzerland), and Mn (Fluka, Buchs, Switzerland) were used. The detector dwell time was 100 μs, the repetition was 3 times. The measured isotopes were ^55^Mn, ^56^Fe and ^59^Co using He as collision gas and ^45^Sc and ^165^Ho (all used modes) as internal standards.

The microwave-assisted syntheses of nanoparticles were carried out using an Anton Paar Monowave 450 microwave system equipped with a MAS24 autosampler (Anton Paar GmbH, Graz, Austria). The system has a maximum power capacity of 850 W, and the required power for each synthesis will be adjusted according to the programmed temperature. The pressure and temperature could be simultaneously monitored by a built-in infrared sensor (IR) during the reaction. The G30 vials (30 mL borosilicate vials) were used as reaction vessels.

Heating efficiency of the nanoparticles was evaluated using an induction heating furnace (Trumpf Hüttinger, Truheat HF 5010, Freiburg, Germany) equipped with water cooled copper heating coils of 40 mm diameter and 5 number of turns. For low-heating-efficiency particles, temperature differences are negligible at lower concentrations, so a 5 mg mL^−1^ concentration was chosen for accurate comparison of the systems. For sample preparation, vacuum-dried particles were transferred into an isolated double-walled glass vessel and dispersed in constant volume of toluene (1 mL) followed by 2–5 min ultrasonication to ensure the homogeneity of the mixture. The vessel was positioned in the axial and radial center of the coil and measured for 10 min under the application of magnetic field at a fixed frequency of 297 kHz, maximum power of 5.55 kW and an applied current of 29.7 A. The magnetic field strength (*H*) was calculated from the following equation:2
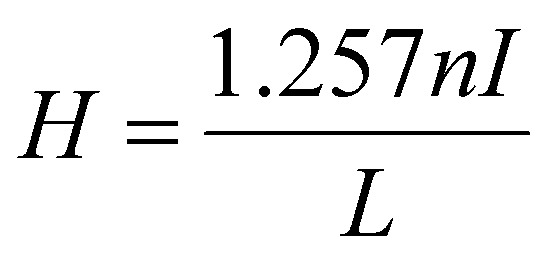
Here, *n* is the number of heating coil turns, *I* is the applied current and *L* is the diameter of coil in centimeters.^38^ Employing the above equation, the calculated value for the magnetic field strength was 46.67 Oe (equivalent to 3.71 kA m^−1^).

Under appliance of the field, the temperature of the magnetic fluid was monitored every 10 s using a radio frequency fiber optic temperature sensor (TS3, Weidmann Technologies GmbH, Dresden, Germany). Based on the recorded data, the heating efficiency of the particles was evaluated as the specific absorption rate (SAR) with values calculated in (W g_NP_^−1^) according to the following equation:3
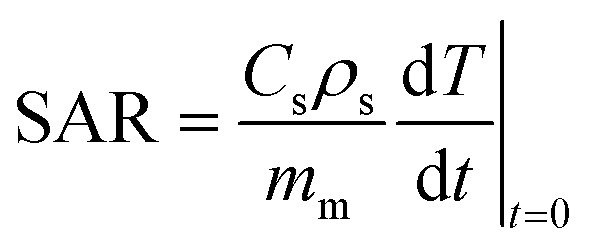
Here, *c*_s_ and *ρ*_s_ are the specific heat capacity of the solvent and density of the sample, respectively. *m*_m_ signifies the weight of the magnetic phase of the nanoparticles per mL of the sample.

Field dependent magnetic measurements were performed on oleic acid coated cobalt ferrite nanoparticles. Powders of the different nanoparticle samples were loaded in PE capsules, which were attached to the sample holder rod of a Vibrating Sample Magnetometer (VSM) for measuring the magnetization *M*(*H*) in a Quantum Design Physical Property Measurement System (PPMS DynaCool, Quantum Design, San Diego, USA). For all samples, the isothermal magnetization was determined in a full hysteresis loop (0 → +H → −H → +H) at temperatures of 2 and 300 K with applied external magnetic fields of up to 90 kOe (70 687 kA m^−1^). The mass susceptibility (*χ*_*ρ*_) associated with the magnetic phase of the nanoparticles was evaluated by subtracting the ligand surface coverage, as calculated from elemental analysis data, from the total mass of the nanoparticles used in each measurement.

## Syntheses

### Synthesis of superparamagnetic M_*x*_Fe_3−*x*_O_4_ (M = Mn, Fe, Co) nanoparticles using thermal decomposition method (TD)

The synthesis of superparamagnetic MFe_2_O_4_ (M = Mn, Fe, Co) nanoparticles was carried out based on a modified procedure published by Sun *et al.*^30^ Briefly, 3.53 g (10 mmol) iron(iii) acetylacetonate, 10.12 g (50 mmol) 1,2-dodecandiol, 10 mL oleic acid and 10 mL oleyl amine were dissolved in 100 mL benzyl ether and stirred at 500 rpm under a flow of argon. The reaction mixture was heated to 200 °C and held at this temperature for 30 min, then heated to 300 °C and held for an additional 30 min. The resulting black-brown mixture was cooled to room temperature. The particles were magnetically decanted and washed 3 times with 100 mL ethanol. The oleic acid-functionalized magnetite nanoparticles were designated as TD_OA@Fe_3_O_4_.

Ferrite nanoparticles were synthesized applying the same reaction conditions as above but using a 1 : 2 molar ratio of Co(acac)_2_ : Fe(acac)_3_ or Mn(acac)_2_ : Fe(acac)_3_ and named TD_OA@Co_*y*_Fe_3−*y*_O_4_ and TD_OA@Mn_*x*_Fe_3−*x*_O_4_ (see ESI†).

### Microwave-assisted synthesis of superparamagnetic M_*x*_Fe_3−*x*_O_4_ (M = Mn, Fe, Co) nanoparticles_method 1 (MW1)

Magnetic nanoparticles were produced using a microwave-assisted method based on the ratio used for thermal decomposition reactions. Therefore, for synthesis of magnetite nanoparticles, 0.265 g (0.75 mmol) iron(iii) acetylacetonate, 0.759 g (3.75 mmol) 1,2-dodecandiol, 0.71 mL (2.25 mmol) oleic acid and 0.74 mL (2.25 mmol) oleyl amine were dissolved in 7.5 mL benzyl ether in a 30 mL microwave vial and magnetically stirred for 10 min before the reaction. The sample was placed in the microwave and heated up to 200 °C in 30 min and held at the same temperature for 10 min. The sample was subsequently heated to 250 °C in 30 min and kept at this temperature for another 5 min. The resulting mixture was centrifuged and washed with ethanol 3 times to remove the unreacted organic residue and labeled as MW1_OA@Fe_3_O_4_.

Similarly, the other metal ferrite nanoparticles were synthesized by the first microwave-assisted method (MW1) using a 1 : 2 molar ratio of the metal precursors and named MW1_OA@Co_*y*_Fe_3−*y*_O_4_ and MW1_OA@Mn_*x*_Fe_3−*x*_O_4_ (see ESI†).

### Synthesis of superparamagnetic Co_*y*_Fe_3−*y*_O_4_ nanoparticles using microwave-assisted method_method 2 (MW2)

Co_*y*_Fe_3−*y*_O_4_ nanoparticles were produced using a microwave-assisted method based on a modified literature procedure.^39^ For synthesis of MW2_NP1 to MW2_NP5 samples, 0.064 g (0.25 mmol) cobalt(ii) acetylacetonate, 0.176 g (0.5 mmol) iron(iii) acetylacetonate, 3 mL oleic acid and 7.5 mL oleyl amine were mixed with 1.5 mL benzyl ether in 30 mL microwave vials (G30) and magnetically stirred at room temperature for 10 min to provide homogeneous dark red mixtures. For the synthesis of MW2_NP5_1Diol to MW2_NP5_5Diol samples, various molar ratios of 1,2-dodecandiol with respect to the molar amounts of precursors (Table S1†) were mixed with similar molar amounts of above-mentioned chemicals in G30 vials and stirred at the same conditions to provide homogeneous mixtures. Oleic acid coated particles were synthesized applying different heating rate and temperatures and were then purified by washing with ethanol (see ESI†).

## Results and discussion

### Superparamagnetic M_*x*_Fe_3−*x*_O_4_ (M = Mn, Fe, Co) nanoparticles (TD, MW1)

**Scheme 1:**
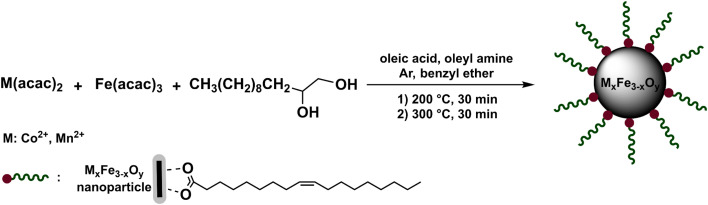
Synthesis of ferrites by thermal decomposition of organic precursors.

Magnetite nanoparticles can be synthesized *via* a thermal decomposition (TD) method delivering highly homogeneous particles with a narrow size distribution. These uniform nanoparticles are crucial for precise control in induction heating and serve as excellent precursors for further surface functionalization, as demonstrated in previous studies.^14^ This method has also been adapted for the synthesis of ferrites by decomposing mixtures of Fe(acac)_3_, Co(acac)_2_ and Mn(acac)_2_ in the presence of 1,2-dodecandiol, oleic acid, and oleyl amine in benzyl ether (Scheme 1).

In a first step, we systematically compared the syntheses of ferrites with Fe, Mn and Co as the metal ions in the oxidation state +II produced by both thermal decomposition and microwave-assisted synthesis. In these syntheses oleic acid serves as a surfactant, ensuring the production of stable, monodisperse nanoparticles, while oleyl amine functions both as a stabilizer and a reducing agent.^46^ In addition, 1,2-dodecanediol acts primarily as a reducing agent, facilitating the conversion of Fe^3+^ to Fe^2+^ ions, particularly in the formation of Fe_3_O_4_ nanoparticles. Subsequently, the TD method was also used as the basis for the synthesis of the same particles in the microwave (MW1 method), which might have the advantage of producing particles with more uniform sizes due to homogeneous nucleation in the microwave system. Hence, similar molar ratios of reactants were used for synthesis of the particles in the microwave system within two heating steps at 200 and 250 °C.

### Structure, morphology and size distribution (TD, MW1)

**Fig. 1:**
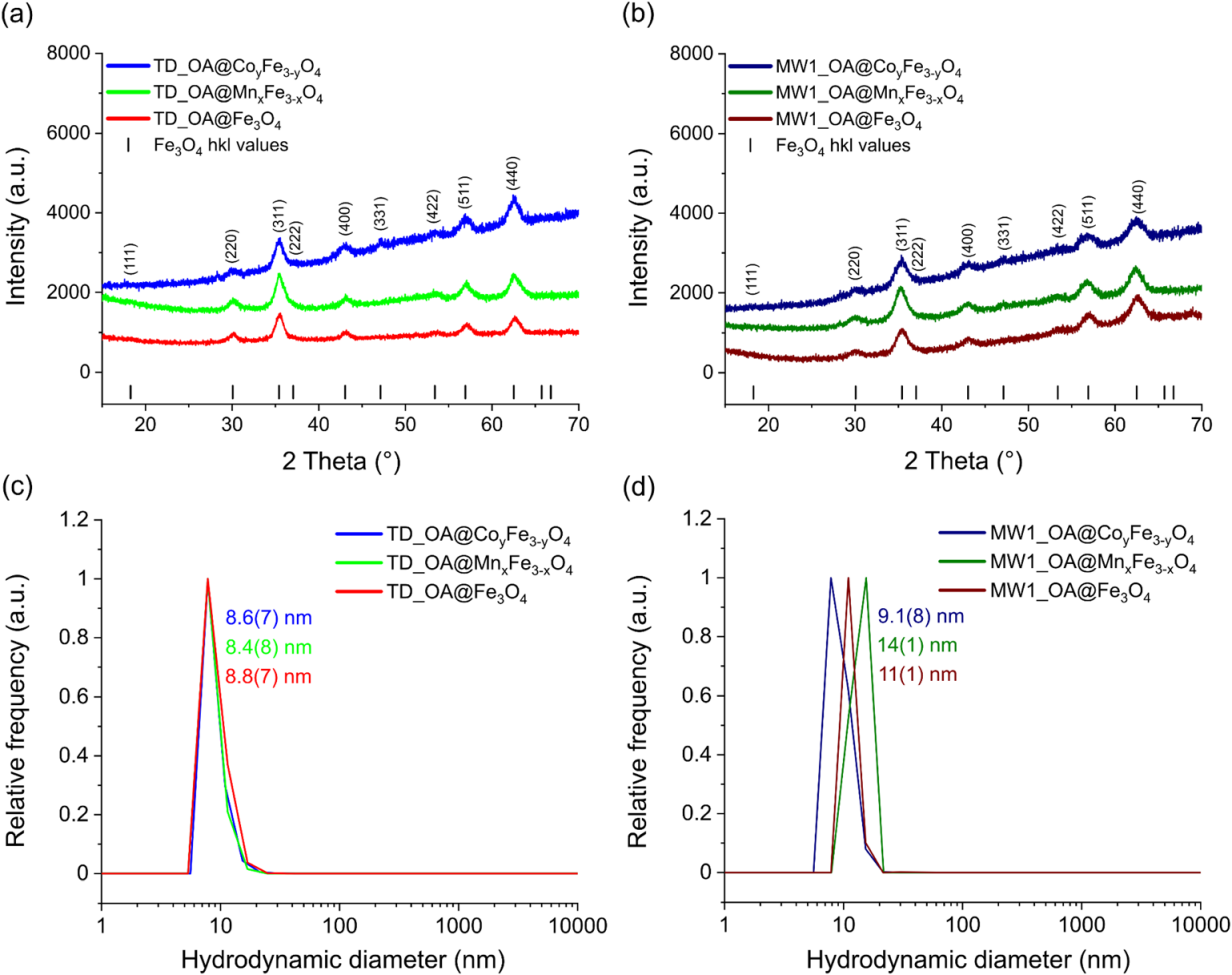
PXRD diffraction patterns of the particles synthesized with (a) TD method and (b) MW1 method in comparison with the Fe_3_O_4_ reference structure^50^ and their respective DLS measurements (c) and (d) measured in *n*-hexane.

Powder X-ray diffraction (PXRD) and dynamic light scattering (DLS) were used to determine the crystalline structure and hydrodynamic diameter of the synthesized oleic acid coated particles (Fig. 1a–d). The diffraction patterns for the magnetite, cobalt ferrite, and manganese ferrite nanoparticles exhibit reflections consistent with the inverse spinel structure, as reported in the literature, with no evidence of impurities.^47,48^ In the case of manganese and cobalt ferrite nanoparticles, Fe^2+^ ions are expected to be replaced by Mn^2+^ and Co^2+^ ions, maintaining the overall spinel structure. The gradual increase in the baseline of the diffractograms at higher 2*θ* angles is attributed to the fluorescence effect of metal ions, which becomes more prominent with higher Co^2+^ or Mn^2+^ content, particularly in samples synthesized *via* the MW1 method compared to the TD method (Fig. 1a and b).

Despite the larger average hydrodynamic diameter of the nanoparticles synthesized *via* the MW1 method, their calculated average crystallite size was slightly smaller compared to those produced using the TD method (Table 2). The average hydrodynamic diameter (*D*_hyd_) of the oleic acid-coated nanoparticles determined by DLS, are consistently larger than the diameters measured by TEM and XRD. The difference arises from the presence of an organic layer of oleic acid on the particle surface and its interactions with the surrounding medium. For nanoparticles synthesized using the MW1 method, *D*_hyd_ is slightly larger than those produced by the TD method. Since the total diameter includes the magnetic core and the oleic acid coating (approximately 2 nm), the hydrodynamic diameter can be estimated as *D*_hyd_ = *D*_TEM_ + 4 nm.^47,49^ This estimation aligns well with the measurements for microwave synthesized particles. However, for nanoparticles synthesized using the TD method, the estimation is less accurate due to a broader size distribution and the presence of some cubic-shaped manganese ferrite nanoparticles. Consequently, the hydrodynamic diameter is only slightly larger than *D*_TEM_ (Table 2).

**Table 2:** Average particle diameter produced with the TD and MW1 methods, determined by XRD (*D*_XRD_), DLS (*D*_hyd_) and TEM (*D*_TEM_)

Sample code	*D*_XRD_ (nm)	*D*_hyd_ (nm)	*D*_TEM_ (nm)
TD_OA@Co_*y*_Fe_3−*y*_O_4_	5.1(1)	8.6(7)	8(2)
TD_OA@Mn_*x*_Fe_3−*x*_O_4_	5.0(1)	8.4(8)	7(2)
TD_OA@Fe_3_O_4_	5.9(1)	8.8(7)	8(2)
MW1_OA@Co_*y*_Fe_3−*y*_O_4_	3.5(1)	9.1(8)	5.8(9)
MW1_OA@Mn_*x*_Fe_3−*x*_O_4_	3.9(1)	14(1)	6(1)
MW1_OA@Fe_3_O_4_	4.1(1)	11(1)	6(1)

**Fig. 2:**
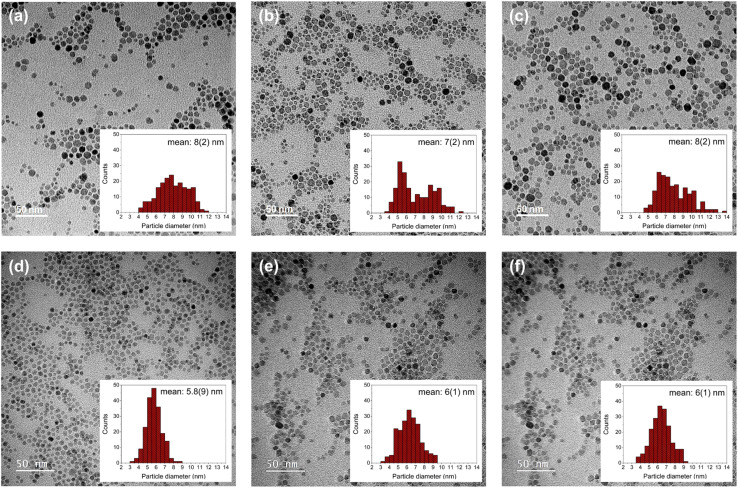
TEM images of the nanoparticles synthesized with the TD method (a) TD_OA@Co_*y*_Fe_3−*y*_O_4_, (b) TD_OA@Mn_*x*_Fe_3−*x*_O_4_ and (c) TD_OA@Fe_3_O_4_ nanoparticles, and particles synthesized with the MW1 method (d) MW1_OA@Co_*y*_Fe_3−*y*_O_4_, (e) MW1_OA@Mn_*x*_Fe_3−*x*_O_4_ and (f) MW1_OA@Fe_3_O_4_ with their respective statistical size distributions (*N* = 200).

TEM images indicate the formation of spherical ferrite nanoparticles with an average diameter of 7 to 8 nm for the ones synthesized with the TD method and 5 to 6 nm for those synthesized with the MW1 method (Fig. 2a–f). The slight size difference can be due to higher temperature (300 °C) applied in particle growth step in the TD method resulting in larger particles.^32^ No agglomeration was detectable in any of the samples. Nanoparticles synthesized with the MW1 method were significantly more uniform in shape and size resulting in narrower size distribution compared to those synthesized with the TD method. This is likely due to more homogeneous nucleation steps in the microwave.^39^ In the case of manganese ferrites produced with TD method, cubic shape nanoparticles were observed beside spherical ones, which is a known feature^48^ and this is not the case for all other samples (Fig. 2b). The mean diameter of the particles calculated from TEM images (*D*_TEM_) were in good agreement with hydrodynamic diameter (*D*_hyd_) of the particles and slightly larger than *D*_XRD_ due to the surface coating with oleic acid and possible amorphous parts in synthesized particles (Table 2).

### Surface coverage (TD, MW1)

Table 3 presents the surface coverage of the particles, calculated based on TG mass losses in the range of 100 to 500 °C, which corresponds to the decomposition temperature of oleic acid, and compared to the values calculated from elemental analysis. The results from both methods were in close agreement, indicating ferrites synthesized with both TD and MW1 methods exhibited higher surface coverage compared to magnetite nanoparticles. The surface coverage calculated from TG mass losses was lower than that obtained from elemental analysis. This deviation is likely due to the partial exclusion of residual carbon from the decomposition of oleic acid below 500 °C, which further converts into CO and CO_2_ during heating under air. This indicates that the surface coverage calculated based on CHN is more accurate compared to the TGA results. Moreover, no nitrogen was detected by elemental analysis indicating that the surface of the particles is only covered with oleic acid (Table 3).

**Table 3:** Surface coverage of particles synthesized with the TD and MW1 method calculated from TG and elemental analysis data

Sample code	TG mass loss (%) 100–500 °C	CHN (%)			Surface coverage (mmol g^−1^)		
		C	H	N	TGA	C	H
TD_OA@Co_*y*_Fe_3−*y*_O_4_	14.68	13.28	2.22	—	0.52	0.61	0.65
TD_OA@Mn_*x*_Fe_3−*x*_O_4_	13.12	15.11	2.41	—	0.46	0.70	0.70
TD_OA@Fe_3_O_4_	8.00	8.30	1.46	—	0.28	0.38	0.43
MW1_OA@Co_*y*_Fe_3−*y*_O_4_	16.74	15.42	2.49	—	0.59	0.71	0.73
MW1_OA@Mn_*x*_Fe_3−*x*_O_4_	11.92	11.17	1.89	—	0.42	0.52	0.55
MW1_OA@Fe_3_O_4_	10.22	11.00	1.88	—	0.36	0.51	0.55

### Chemical composition (TD, MW1)

The chemical composition of the substituted particles was analyzed using ICP-MS and SEM-EDX (Table 4). For the Co_*y*_Fe_3−*y*_O_4_ nanoparticles, the amount of cobalt calculated by both methods was slightly lower than the theoretical ratio. However, in the case of manganese ferrites, only a small amount of manganese was detected in the particles. This could be caused by the delayed initiation of decomposition for Mn(acac)_2_ in the reaction mixture (decomposition temperature 246 °C) in comparison to Fe(acac)_3_ (decomposition temperature 220 °C) resulting in a lower incorporation of manganese compared to iron ions.^51,52^ On the other hand, the initial decomposition temperature for Co(acac)_2_ is estimated to be 183 °C, hence, more iron ions are expected to be replaced by Co^2+^ ions in the structure (Fig. S3†). Furthermore, 1,2-dodecandiol is used as a common reducing agent in thermal decomposition synthesis of ferrite nanoparticles that can reduce the Fe^3+^ ions to Fe^2+^ ions during the synthesis.^30^ This can result in a competition between M^2+^ ions and Fe^2+^ ions and lead to lower contribution of M^2+^ ions in the structure of ferrites.

**Table 4:** Chemical composition of the particles synthesized with the TD and MW1 method, calculated from ICP-MS and EDX analysis data

Sample code	EDX				ICP-MS	
	Fe	Co	Mn	Chemical composition	Fe : M^2+^ (mmol)	Chemical composition
TD_OA@Co_*y*_Fe_3−*y*_O_4_	72.7	27.3	—	Co_0.82_Fe_2.18_O_4_	2.51	Co_0.85_Fe_2.15_O_4_
TD_OA@Mn_*x*_Fe_3−*x*_O_4_	92.1	—	7.9	Mn_0.26_Fe_2.74_O_4_	11.89	Mn_0.23_Fe_2.77_O_4_
MW1_OA@Co_*y*_Fe_3−*y*_O_4_	71.2	28.8	—	Co_0.86_Fe_2.14_O_4_	2.34	Co_0.90_Fe_2.10_O_4_
MW1_OA@Mn_*x*_Fe_3−*x*_O_4_	87.0	—	13.0	Mn_0.39_Fe_2.74_O_4_	6.38	Mn_0.41_Fe_2.59_O_4_

### Heating efficiency in magnetic field (TD, MW1)

**Fig. 3:**
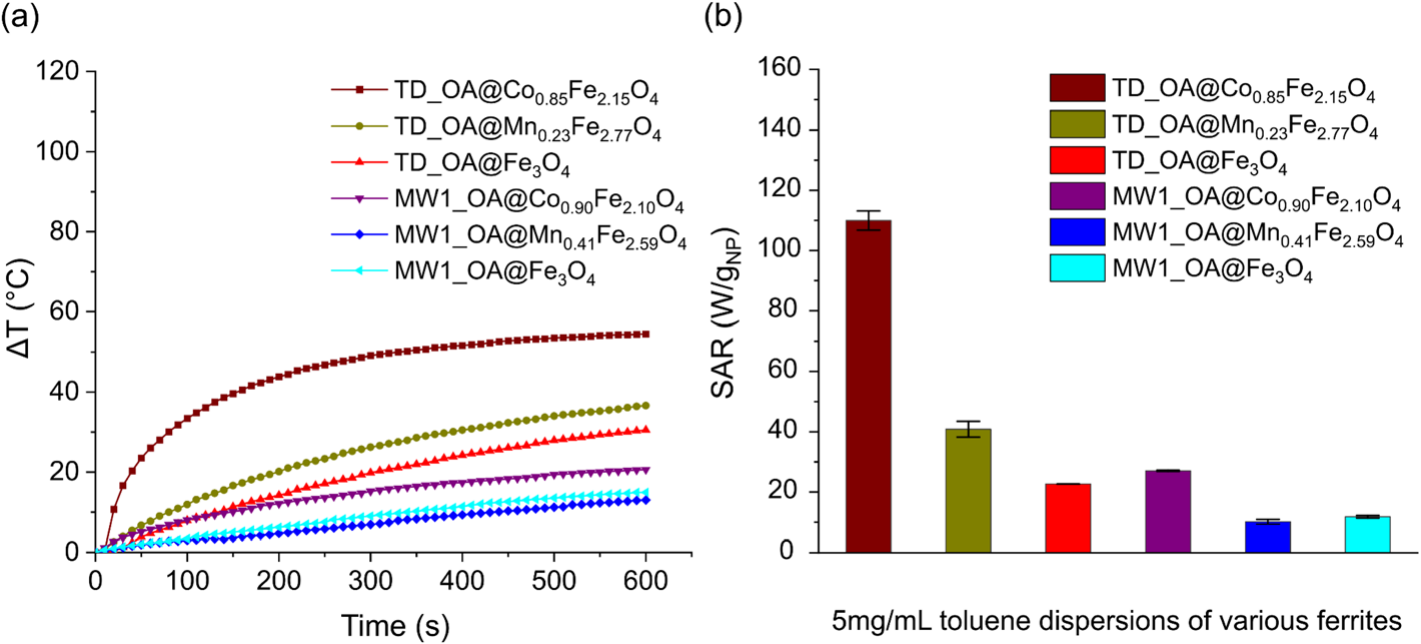
Heating efficiency of particles synthesized with both TD and MW1 method: (a) temperature variation as a function of time and (b) SAR values calculated for 5 mg mL^−1^ of magnetic particle dispersion.

To assess the heating capacity of nanoparticles synthesized *via* both TD and MW1 methods, the temperature variation in a 5 mg mL^−1^ particle dispersion in toluene was monitored under an alternating magnetic field in a closed system (Fig. 3a). Each measurement was conducted 3 times to assure the accuracy of the data. SAR values were calculated applying the slope of time dependent temperature variation curves based on the eqn (3) under non-adiabatic conditions (Fig. 3b). Given the notable slope variation, only the first 2 min of the measurements were considered for the calculation of the SAR values. Nanoparticles synthesized by the TD method showed a larger temperature increase than those synthesized *via* the MW1 method, possibly due to the larger particle sizes achieved *via* TD method. However, the precise cause for the lower heating efficiency of MW1-synthesized particles remains undetermined. For TD-synthesized particles, the heating efficiency of the particles increased with the substitution of Fe^2+^ by manganese and cobalt, consistent with literature; this effect was not observed in MW1-synthesized particles. To verify the stability of the surface coverage after the measurements, particle dispersion was centrifuged at 8000 rpm for several times to separate the particles and the supernatant was measured with FTIR spectroscopy. Only the signals corresponding to toluene were found in the FTIR spectra and no sign of oleic acid or relative moieties was found in the supernatant as an indication that the particle surface coverage is stable (Fig. S4†).

**Fig. 4:**
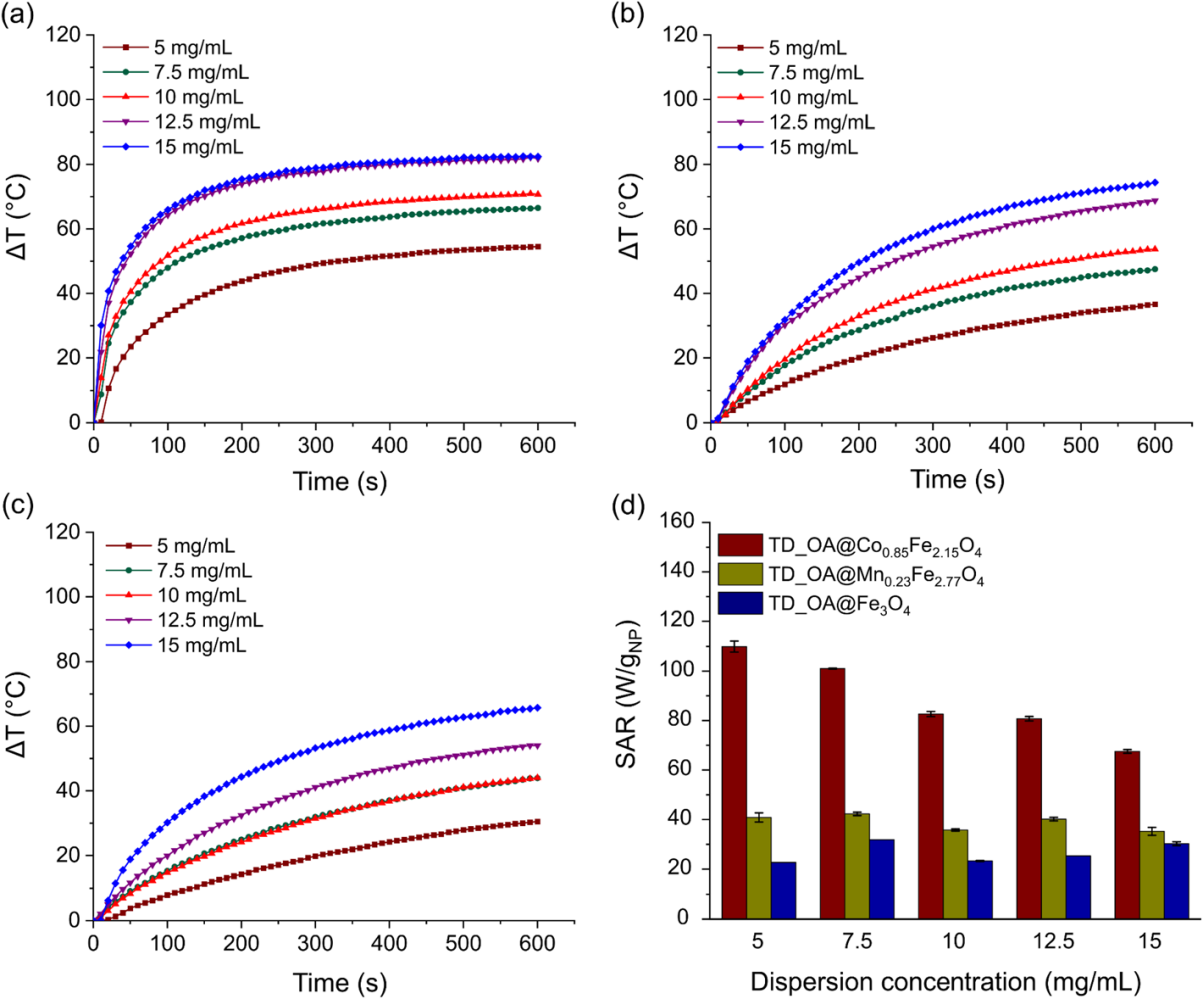
Temperature variation as a function of time for various concentrations of TD-synthesized magnetic particles: (a) TD_OA@Co_0.85_Fe_2.15_O_4_, (b) TD_OA@Mn_0.23_Fe_2.77_O_4_, (c) TD_OA@Fe_3_O_4_ and (d) the respective calculated SAR values.

Furthermore, the impact of particle concentration on the heating rate and SAR value of magnetic particles was investigated for a range of 5 to 15 mg of TD-synthesized particles per mL of toluene (Fig. 4a–d). Under the appliance of the magnetic field, particles generate heat through Néel and/or Brownian relaxation. For all three types of TD-synthesized OA-coated ferrites, increasing the particle amount from 5 to 15 mg at constant field parameters (amplitude and frequency) leads to higher temperatures. At similar concentrations, TD_OA@Co_0.85_Fe_2.15_O_4_ showed the highest heating efficiency, while magnetite particles exhibited the lowest heating rate among all samples. The heat generated by TD_OA@Mn_0.23_Fe_2.77_O_4_ particles at different concentrations was slightly higher than that of magnetite particles, which is most likely due to the low substitution degree of Fe^2+^ ions by Mn^2+^ ions. These findings suggest that even low substitution of magnetite with Mn^2+^, Co^2+^ ions can significantly affect the properties, particularly the heating efficiency of the particles.

Due to the different degree of surface coverage of the TD-synthesized ferrites, a precise comparison of the heating efficiency of the particles based on temperature variation alone is not possible. Therefore, to obtain a better evaluation, SAR values were calculated based on the mass of the magnetic phase of the particles using eqn (3). Furthermore, due to the variation of the slope of the temperature variation per time curve, the initial slope (temperature variation within 120 s) was considered in the SAR calculation. Fig. 4d shows that the highest calculated SAR value was about 110 W g_NP_^−1^ for 5 mg mL^−1^ toluene dispersion of TD_OA@Co_0.85_Fe_2.15_O_4_ and decreases with increasing particle concentration. The increase of the particle concentration results in a decrease of interparticle distances leading to enhancement of interparticle interactions, reduction of anisotropy barrier and Néel relaxation time and eventually decrease of the SAR value.^15,53^ The deviation of the calculated SAR when increasing the particle concentration for magnetite and manganese ferrite can be due to possible formation of linear chain-like structures that form at high concentrations of magnetic dispersions and lead to a lower response of the particles in the magnetic field.^15^

### Superparamagnetic Co_*y*_Fe_3−*y*_O_4_ nanoparticles (MW2)

In a subsequent approach, the focus was narrowed to cobalt ferrite nanoparticles synthesized by the TD method, identified as the best performing superparamagnetic particles from the first systematic study, to evaluate the advantages and limitations of different synthesis methods for a single particle type. Additional syntheses of cobalt ferrite nanoparticles with varying ratios of 1,2-dodecandiol were performed to elucidate the effect of the diol on particle properties. In this series, the MW2 method was used to prepare cobalt ferrite particles with the general formula of Co_*y*_Fe_3−*y*_O_4_ under varying reaction conditions and molar amounts of diol (Tables S1 and S2†). Benzyl ether was used as the solvent to ensure comparability with previous systems and due to its dielectric constant (*ε*_benzyl ether_ = 3.86), which critically influences the microwave field interaction with the reaction mixture. Based on previous findings, a molar ratio of 1 : 2.4 (oleic acid to oleyl amine) was chosen to promote spherical particle morphology.^54,55^ The synthesis of the cobalt ferrite particles involved two distinct heating steps for nucleation and particle growth to achieve uniform particles with narrow size distribution (Table S2†).^33^

Particles obtained from the MW2 method were compared in detail regarding their structure and size distribution, surface coverage, composition and heating efficiency in magnetic field to achieve particles with improved properties.

### Structure, morphology and size distribution (MW2)

**Fig. 5:**
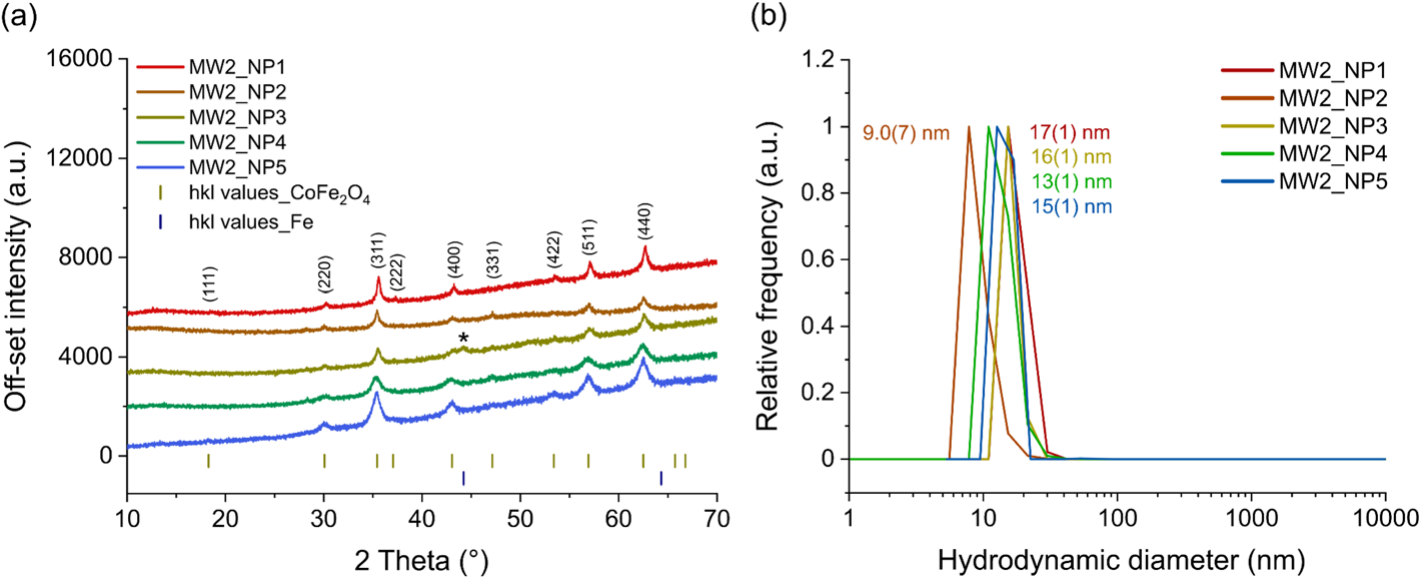
(a) PXRD diffraction patterns of cobalt ferrite nanoparticles synthesized with the MW2 method in comparison with reference structure^50^ and (b) their respective hydrodynamic diameter measured with DLS in *n*-hexane.

Powder X-ray diffraction (PXRD) and dynamic light scattering (DLS) were used to determine the crystalline structure and hydrodynamic diameter of the synthesized oleic acid-coated cobalt ferrite particles (Fig. 5 and S5†). Except for MW2_NP3, the positions of the reflections in the powder diffractogram for all samples correspond to the reflections of the inverse spinel structure of cobalt ferrite as documented in the literature and no additional phases are observed. An additional reflection at 44.2° (*) is detectable for the MW2_NP3 sample as an indication of elemental iron (12 wt% of sample). This could be due to the formation of iron(ii) oxide (FeO) during the reaction as a result of the reduction of Fe^3+^ to Fe^2+^, which occurs in the presence of an excess amount of oleyl amine. The structure of FeO is not chemically stable, therefore, it can be further decomposed to Fe_3_O_4_ and elemental iron (Fig. 5a).^56,57^

**Fig. 6:**
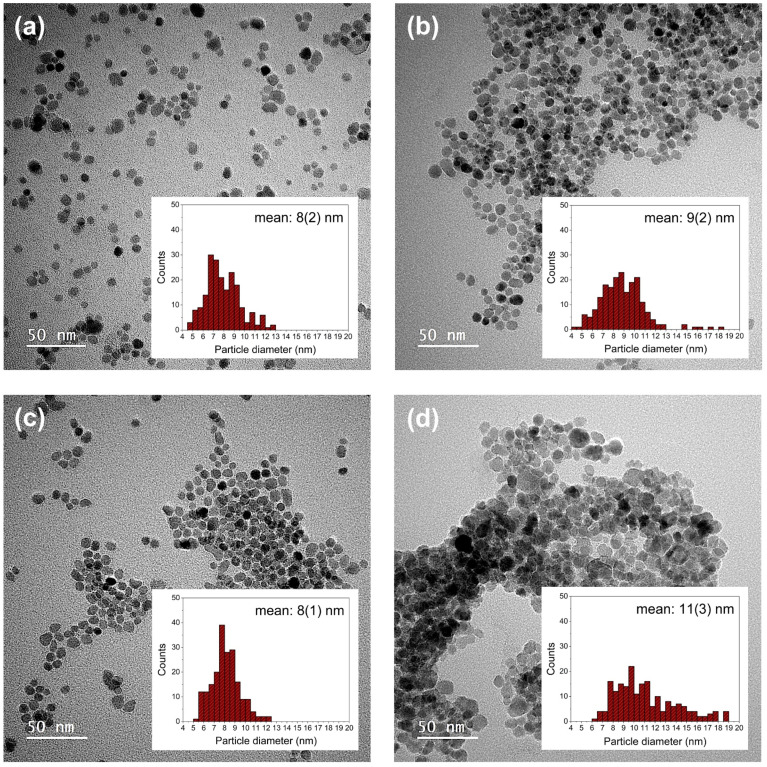
TEM images of particles synthesized with the MW2 method without diol (a) MW2_NP5, and in the presence of various amounts of diol (b) MW2_NP5_1Diol, (c) MW2_NP5_3Diol and (d) MW2_NP5_5Diol with their respective statistical size distributions (*N* = 200).

Reducing the heating rates for both the nucleation and particle growth step leads to a reduction in particle diameter (Table 5), which is consistent with the literature results.^58^ Decreasing the heating rate in nucleation step allows the complete decomposition of the precursors and the formation of more nuclei, leading to the formation of small particles with uniform sizes in the next step. In addition, beyond its reducing effect in the synthesis of ferrites, the diol has an influence on the shape and size of the particles.^51,58^ Therefore, addition of the slight deviations of the particle shapes from the spherical shape in comparison to those produced by the MW1 method could be attributed to the absence of diol in the MW2 method. To validate this hypothesis, particles were synthesized under the same conditions as the MW2_NP5 sample, in the presence of different molar amounts of 1,2-dodecandiol. Fig. 6 illustrates the TEM images of particles synthesized without and with different molar ratios of 1,2-dodecandiol. Nanoparticles synthesized with 1 : 1 molar ratio of precursor to 1,2-dodecandiol (MW2_NP5_1Diol) have an almost spherical shape, but the particles were partially agglomerated. Increasing the molar ratio of diol in MW2_NP5_3Diol resulted in the formation of spherical particles with narrow size distribution, and no evidence of agglomeration was observed. When the molar ratio of 1,2-dodecandiol in MW2_NP5_5Diol was further increased, the particles deviated from spherical shape and showed considerable agglomeration (Fig. 6 and Table 5).

**Table 5:** Average particle diameter of particles synthesized with the MW2 method compared to TD-synthesized cobalt ferrites, determined by XRD (*D*_XRD_), DLS (*D*_hyd_) and TEM (*D*_TEM_)

Sample code	*D*_XRD_ (nm)	*D*_hyd_ (nm)	*D*_TEM_ (nm)
MW2_NP1	13.7(3)	17(1)	N.A.
MW2_NP2	10.2(3)	9.0(7)	N.A.
MW2_NP3	9.4(2)	16(1)	N.A.
MW2_NP4	5.6(1)	13(1)	8(2)
MW2_NP5	6.6(1)	15(1)	8(2)
MW2_NP5_1Diol	13.0(2)	18(2)	9(2)
MW2_NP5_3Diol	7.4(1)	16(1)	8(1)
MW2_NP5_5Diol	9.9(2)	30(1)	11(3)
TD_OA@Co_0.85_Fe_2.15_O_4_	5.1(1)	8.6(7)	8(2)

Results obtained from DLS and PXRD support the TEM observations showing larger crystallite sizes and also hydrodynamic diameters in samples produced with 1 : 1 and 1 : 5 molar ratio of precursor to diol (Table 5 and Fig. S5†). This can be explained by the role of diol as a reducing agent and its ability to increase the nucleation rate which leads to the formation of a large number of nuclei. This, in turn, enhances the production of metal monomers, resulting in higher supersaturation^59^ and subsequently limiting particle growth, which explains the narrower size distributions observed in the presence of diol. However, while this effect is beneficial for size control, the rapid formation of a large number of nuclei also raises the risk of particle agglomeration (Fig. 6).

### Surface coverage and chemical composition (MW2)

Surface coverage of the particles was evaluated using FTIR and quantified based on TG and elemental analysis data (Fig. S6† and Table 6). The TG mass loss in the range of 100 to 500 °C was used to determine surface coverage, ensuring that the observed mass loss was solely due to oleic acid decomposition. Elemental analysis data indicated a small presence of nitrogen in samples synthesized *via* the MW2 method without diol, suggesting potential oleyl amine bonding on the particle surface. To calculate oleic acid surface coverage based on elemental analysis data, the contribution of oleyl amine was first isolated by determining the nitrogen content detected in each sample. The carbon content associated with oleyl amine was then calculated and subtracted from the total carbon percentage detected in the sample, allowing for the determination of the carbon percentage attributed to oleic acid. A similar approach was employed for calculating the hydrogen percentages. Finally, the surface coverage by oleic acid was estimated using the recalculated carbon and hydrogen values. Among samples prepared with the MW2 method in the absence of diol, MW2_NP4 and MW2_NP5 samples showed relatively high surface coverage (0.50 and 0.43 mmol g^−1^, respectively) with both oleic acid and oleyl amine. In contrast, MW2_NP1 to MW2_NP3 samples, displayed much lower surface coverage which could be due to the high pressure of gases during the reaction (Fig. S6† and Table 6). During the particle syntheses, volatile contents, mainly CO and CO_2_, will form due to the decomposition of precursor and solvent at high temperatures. In a system with an inert atmosphere the gases will be transferred with the gas flow preventing the particles from being affected by these species. However, in a closed system such as a microwave, these fragments and volatile contents will remain in the vessel, influencing the particles by bonding to the surface of nuclei after formation. This would prevent the bonding of other ligands, such as oleic acid and oleyl amine, to the surface of the particles or can affect the final shape of the particles by acting as a stabilizer for the nuclei.^55,60,61^ Hence, optimized reaction conditions used for sample MW2_NP5, can effectively prevent the high pressure during the reaction and resulting in high surface coverage. The total surface coverage calculated from TGA data was in good agreement with the total surface coverage of both oleic acid and oleyl amine calculated from elemental analysis (Table 6). Addition of the different amounts of the 1,2-dodecandiol to the samples leads to a decrease in surface coverage, in particular for the MW2_NP5_1Diol and MW2_NP5_5Diol samples, due to the agglomeration (Fig. S6† and Table 6).

**Table 6:** Comparison of surface coverage of cobalt ferrite particles synthesized with the MW2 and TD method calculated from TG and elemental analysis data

Sample code	TG mass loss (%) 100–500 °C	CHN (%)			Surface coverage (mmol g^−1^)			
		C	H	N	TGA	C_OA_	H_OA_	N_OAm_
MW2_NP1	6.70	6.16	1.17	0.19	0.24	0.15	0.19	0.14
MW2_NP2	5.18	3.94	0.81	0.10	0.18	0.11	0.16	0.07
MW2_NP3	2.03	3.27	0.67	0.10	0.07	0.08	0.12	0.07
MW2_NP4	11.93	10.92	1.93	0.20	0.42	0.36	0.41	0.14
MW2_NP5	11.42	9.27	1.78	0.29	0.40	0.22	0.29	0.21
MW2_NP5_1Diol	5.82	3.55	0.76	—	0.21	0.16	0.22	—
MW2_NP5_3Diol	9.82	8.49	1.55	0.21	0.35	0.24	0.29	0.15
MW2_NP5_5Diol	6.02	4.77	0.92	—	0.21	0.22	0.27	—
TD_OA@Co_0.85_Fe_2.15_O_4_	14.68	13.28	2.22	—	0.52	0.61	0.65	—

The ICP-MS and EDX results were in a close agreement, demonstrating that the amount of cobalt in the particles were slightly lower than the theoretical composition and, in most cases, were higher than the cobalt content in TD-synthesized cobalt ferrite suggesting a better control over the composition of particles in the MW method in comparison to the TD method. In addition, it was shown that the addition of the diol as the reducing agent had a negligible influence on the cobalt content most likely due to the fact that Co(acac)_2_ decomposes in lower temperatures compared to Fe(acac)_3_, resulting in an early incorporation of the cobalt ions in the structure, however, it might not be the case for the other precursors such as Mn(acac)_2_ and Ni(acac)_2_ (Table S4†).

### Heating efficiency (MW2)

**Fig. 7:**
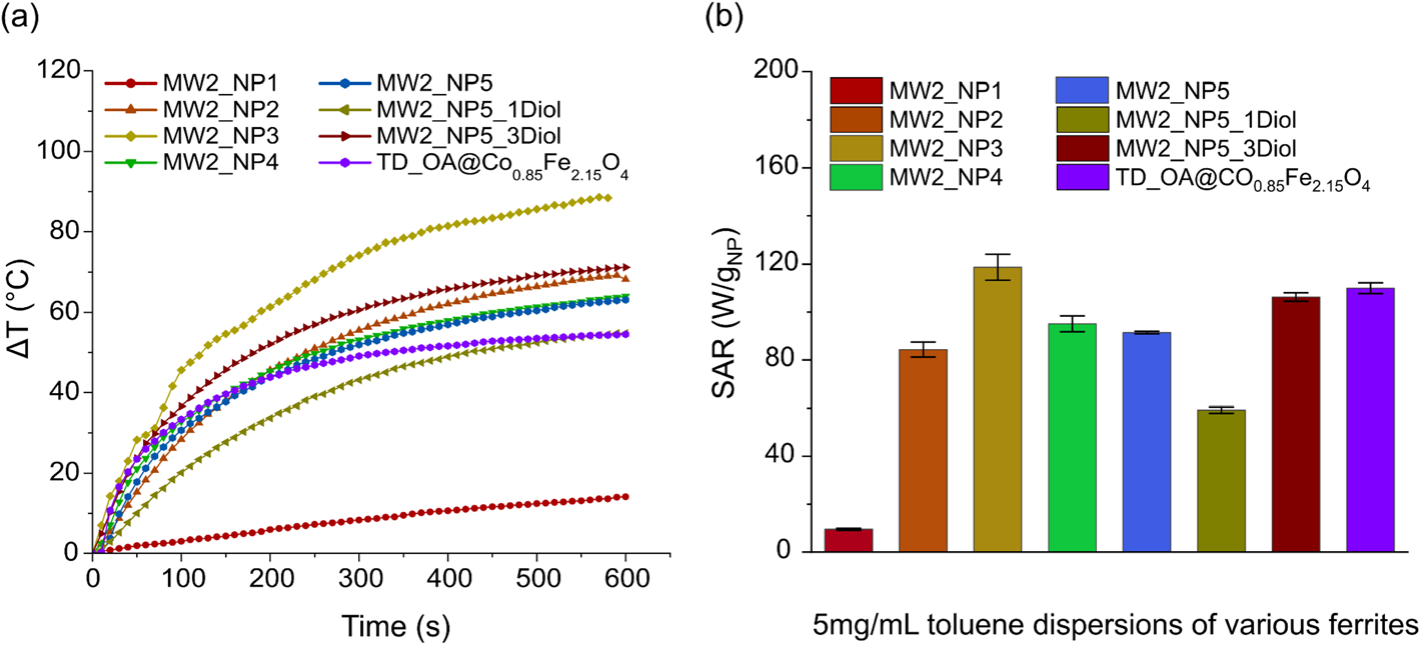
Heating efficiency of particles synthesized with the MW2 method: (a) temperature variation as a function of time and (b) SAR values measured for 5 mg mL^−1^ of magnetic cobalt ferrite particle dispersion.

The heating capacity of the particles synthesized *via* the MW2 method were determined by monitoring the temperature variation of the 5 mg mL^−1^ particle dispersion in toluene under the appliance of a magnetic field in a close system (Fig. 7a). SAR values were calculated using the slope of a time dependent temperature variation curves based on the eqn (3) in non-adiabatic conditions (Fig. 7b). As already described above, only the first 2 min of the measurements were considered for the calculation of the SAR values. Due to the low surface coverage of the particles from samples MW2_NP2 and MW2_NP3, the particles were barely dispersible in toluene. This leads to a more pronounced interaction of the particles during the induction measurement and enhancement of the temperature increase due to the change in the magnetic properties of the particles. In contrast, the particles synthesized at 250 °C had a high surface coverage, providing stable particle dispersion in non-polar solvents such as toluene. 5 mg mL^−1^ dispersions of these samples (MW2_NP4, MW2_NP5) reached a temperature rise of almost 60 °C within 10 min, which is higher than the TD-synthesized sample. In the case of samples synthesized in the presence of diol, increasing the ratio of diol in the reaction from 1 to 3 led to an increase in heating efficiency of the particles most likely due to the higher uniformity of the particles. Therefore, sample MW2_NP5_3Diol demonstrated a temperature rise of almost 71 °C within 10 min, which was considerably higher than the temperature reached for the sample produced with TD method. Particle behavior was analyzed in poly(dimethylsiloxane) of varying viscosities, indicating similar behavior in low-viscosity non-polar polymers and suggesting Néel relaxation as the main heating mechanism (Fig. S7a†). Sample MW2_NP5_5Diol synthesized with higher amount of diol showed very poor dispersibility in the toluene due to the large agglomeration of the particles, making the sample unsuitable for heating efficiency measurements (Fig. 7a and b).

### Magnetic properties

**Fig. 8:**
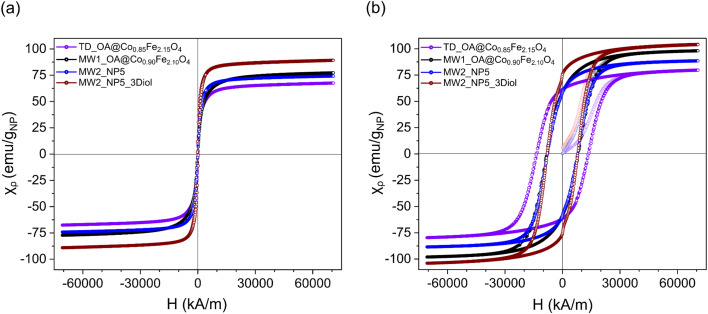
Hysteresis *M*(*H*) curves measured for oleic acid coated cobalt ferrite nanoparticles at (a) 300 K and (b) 2 K.

The magnetic properties of the oleic acid coated cobalt ferrite nanoparticles with optimized properties were measured as full isothermal hysteresis loops at 2 and 300 K (Fig. 8a and b). For each sample, the mass susceptibility was evaluated based on the particle mass after subtracting the oleic acid surface coating. This ensured accurate determination of the particle magnetization and allowed for comparable results regarding the heating efficiency of the particles. The saturation magnetization and coercivity values were extracted from the hysteresis loops for each sample and compared to the determined SAR values (Table 7). At 300 K, all samples exhibited superparamagnetic behavior and high saturation magnetization (*M*_s_), attributed to high cobalt substitution in the structure. At 2 K, which is below the Curie temperature of the particles, all samples displayed ferromagnetic behavior and high coercivity (*H*_c_). This behavior is explained by the low temperature reducing the anisotropy energy required for the magnetic moments to flip, resulting in the moments remaining parallel and exhibiting ferromagnetic properties.^62^

**Table 7:** Magnetic parameters measured for optimized oleic acid coated cobalt ferrite nanoparticles

Sample code	*T* = 300 K		*T* = 2 K		SAR (W g_NP_^−1^)
	*M*_s_ (emu g_NP_^−1^)	*H*_c_ (kA m^−1^)	*M*_s_ (emu g_NP_^−1^)	*H*_c_ (kA m^−1^)	
TD_OA@Co_0.85_Fe_2.15_O_4_	67.5	0.18	79.2	13 750	110.0
MW1_OA@Co_0.90_Fe_2.10_O_4_	77.3	25	98	8085	26.9
MW2_NP5	74.2	117	88.6	7970	90.9
MW2_NP5_3Diol	89.2	242	104.2	8056	106.3

The saturation magnetization values measured at 300 K for all samples generally aligned with the SAR values, except for the sample synthesized using the MW1 method (MW1_OA@Co_0.90_Fe_2.10_O_4_). This discrepancy may be attributed to surface disorder, which increases in smaller particles due to their higher surface-to-volume ratio (Table S5†).^63^ Additionally, the surface coverage of these particles was relatively high (0.71 mmol g^−1^). With a smaller particle volume, the density of the ligands on the surface increases, leading to a decrease in SAR value due to an increase in Brownian relaxation time as a result of an increase in hydrodynamic volume.^14^ Since the mass of particles used for SAR measurements was constant (5 mg), a smaller particle size corresponds to a larger number of particles in the dispersion. These particles with a high surface coverage are likely to interact more strongly with each other and with the surrounding solvent. Such interactions may hinder particle rotation when Brownian relaxation is involved. Overall, sample MW2_NP5_3Diol exhibited the highest saturation magnetization (89.2 emu g^−1^) at 300 K, which is consistent with its superior heating efficiency (106.3 W g_NP_^−1^) in the induction field (Table 7).

## Conclusion

In this study, magnetic ferrite nanoparticles with the general composition M_*x*_Fe_3−*x*_O_4_ (M = Mn, Fe, Co) were synthesized by thermal decomposition (TD) and microwave-assisted (MW) methods. The TD method produced nanoparticles with small sizes (average *D*_TEM_ = 7–8 nm) that exhibited high heating efficiencies in the alternating magnetic field. However, due to non-uniform heating and the influence of diol as a reducing agent, this method respectively resulted in particles with broad size distribution (*D*_TEM_ = 4 to 11 nm) and low degree of heterometal incorporation in the ferrite particles, particularly for manganese. To overcome these problems, a microwave assisted method was used, which increased the amount of Co and Mn incorporation and produced more uniform particles. An optimized microwave-assisted synthesis was further developed to eliminate the reducing effects of the diol reagents and improve the synthesis of cobalt ferrite particles. Using this method, Co_*x*_Fe_3−*x*_O_4_ nanoparticles with a high degree of heterometal content (*x* ≥ 0.85), significant temperature increase (up to 70 °C) under alternating magnetic fields, and remarkable high magnetization (74–89 emu g_NP_^−1^) were successfully synthesized, making them a suitable candidate to be used as heating agent in material science and hyperthermia applications.

## Author contributions

K. M.: conceptualization, investigation, synthesis, characterization, formal analysis, methodology, writing original draft, visualization; L. S.: magnetic measurements, review and editing; R. P.: review and editing; G. K.: conceptualization, funding acquisition, supervision, project administration, resources, writing, review and editing. All authors have read and agreed to the published version of the manuscript.

## Conflicts of interest

There are no conflicts to declare.

## Supplementary Material

NA-OLF-D5NA00244C-s001

## Data Availability

The additional data supporting this article (PXRD, FTIR, TGA, *etc.*) have been included as a part of ESI.†
