# Supplementary material for: Optimizing superparamagnetic ferrite nanoparticles: microwave-assisted vs. thermal decomposition synthesis methods
Source: Nanoscale Adv. 2025 May 26;7(15):4563–76. doi: 10.1039/d5na00244c (PMC12168923; doi:10.1039/d5na00244c)
Supplement: NA-007-D5NA00244C-s001 [file NA-007-D5NA00244C-s001.pdf]

Supplementary Information (SI) for Nanoscale Advances.
This journal is © The Royal Society of Chemistry 2025

Supplementary Information

Optimizing Superparamagnetic Ferrite Nanoparticles: Microwave-Assisted vs. Thermal Decomposition Synthesis Methods

Kimia Moghaddari[a] (ORCID ID: 0000-0001-5726-5980), Lars Schumacher[b] (ORCID ID: 0009-0009-9444-8773), Rainer Pöttgen[b] (ORCID ID: 0000-0003-0962-279X), and Guido Kickelbick[a], [c] * (ORCID ID: 0000-0001-6813-9269)

[a] Inorganic Solid State Chemistry, Saarland University, Campus, Building C4 1, 66123 Saarbrücken, Germany.

[b] Institut für Anorganische und Analytische Chemie, Universität Münster, Corrensstrasse 30, 48149 Münster, Germany.

[c] Saarene – Saarland Center for Energy Materials and Sustainability, Campus C4 2, 66123 Saarbrücken, Germany.

*Corresponding author: guido.kickelbick@uni-saarland.de, Tel.: +49-681-302-70651

Contents

Thermal decomposition (TD) synthesis procedure of nanoparticles 3

Microwave-assisted synthesis procedure of nanoparticles_ Method 1 (MW1) 4

Microwave-assisted synthesis procedure of nanoparticles_ Method 2 (MW2) 5

FTIR measurements of nanoparticles synthesized with TD and MW1 methods 7

TGA measurments of nanoparticles synthesized with TD and MW1 methods 8

Decomposition temperature of molecular precursored measured with TGA................................10

Analysis of supernatant of particle dispersion after heating efficiency measurement 11

PXRD and DLS of cobalt ferrite nanoparticles synthesized with MW2 method in the presence of 1,2-dodecandiol 11

FTIR measurements of cobalt ferrite nanoparticles synthesized with MW2 method 12

Chemical composition of the cobalt ferrite particles synthesized with MW2 and TD method. 12

Impacts of dispersion medium and particle concentration on the heating efficiency 13

Surface to volume ratio of cobalt ferrite particles with optimized properties...............................14

Synthesis of superparamagnetic M_xFe$_{3-x}$O$_4$ (M = Mn, Fe, Co) nanoparticles using thermal decomposition method (TD)

The synthesis of superparamagnetic MFe$_2$O$_4$ (M = Mn, Fe, Co) nanoparticles was carried out based on a modified procedure published by Sun *et al.*[1] In a 500 mL 3-neck round bottom flask 3.53 g (10 mmol) iron (III) acetylacetonate, 10.12 g (50 mmol) 1,2-dodecandiol, 10 mL oleic acid and 10 mL oleyl amine were dissolved in 100 mL benzyl ether and magnetically stirred with a stirring rate of 500 rpm under a flow of argon. The reaction mixture was heated to 200 °C and held at this temperature for 30 min, then heated to 300 °C and held for an additional 30 min. The resulting black-brown mixture was cooled to room temperature. The particles were magnetically decanted and washed 3 times with 100 mL ethanol. The magnetite nanoparticles surface-functionalized with oleic acid were designated as TD_OA@Fe_3O_4 and stored under 90 mL ethanol to avoid any oxidation. To calculate the yield, 2 mL of the dispersion was dried under vacuum and weighed (17.8 mg/2 mL, 801 mg total mass, TGA residual mass after N_2 segment: 86.30%, CHN_{exp}: C: 8.30%, H: 1.46%). Yield: 93%, black solid.

Ferrite nanoparticles were synthesized applying the same reaction conditions as above but using a 1:2 molar ratio of Co(acac)$_2$:Fe(acac)$_3$ or Mn(acac)$_2$:Fe(acac)$_3$. The mixed metal ferrite nanoparticles were resulting from this thermal decomposition route are named TD_OA@$Co_yFe_{3-y}O_4$ and TD_OA@$Mn_xFe_{3-x}O_4$. The synthesis of cobalt ferrite nanoparticles yielded about 93% (18.1 mg/2 mL, 814.5 mg total mass, TGA residual mass after N_2 segment: 74.26%, CHN_{exp}: C: 13.28%, H: 2.22%, experimental chemical formula as obtained by ICP-MS: $Co_{0.85}Fe_{2.15}O_4$) while the calculated yield for manganese ferrite was about 95% (14.7 mg/2 mL, 661.5 mg total mass, TGA residual mass after N_2 segment: 75.14%, CHN_{exp}: C: 15.11%, H: 2.41%, experimental chemical formula as obtained by ICP-MS: $Mn_{0.23}Fe_{2.77}O_4$).

Microwave-assisted synthesis of superparamagnetic M_xFe$_{3-x}$O$_4$ (M = Mn, Fe, Co) nanoparticles_ Method 1 (MW1)

Magnetic nanoparticles were produced using a microwave-assisted method based on the ratio used for thermal decomposition reactions. Therefore, for synthesis of magnetite nanoparticles, 0.265 g (0.75 mmol) iron (III) acetylacetonate, 0.759 g (3.75 mmol) 1,2-dodecandiol, 0.71 mL (2.25 mmol) oleic acid and 0.74 mL (2.25 mmol) oleyl amine were dissolved in 7.5 mL benzyl ether in a 30 mL microwave vial and magnetically stirred for 10 min before the reaction. The sample was placed in the microwave and heated up from room temperature to 200 °C in 30 min and held at the same temperature for 10 min. The sample was subsequently heated to 250 °C for 30 min and kept at this temperature for another 5 min. Finally, the system was cooled to 55 °C and a dark black mixture containing magnetite particles was obtained. To remove the unreacted organic residue, 10 mL ethanol were added to the particle mixture and centrifuged at 7000 rpm for 15 min. The washing step was repeated 3 more times, and finally the oleic acid coated particles were stored under 15 mL ethanol to avoid oxidation and labeled as MW1_OA@Fe_3O_4. To calculate the yield of reaction, about 3 mL of the dispersion was dried under vacuum and weighed (12.2 mg/3 mL, 61 mg total mass, TGA residual mass after N_2 segment: 78.03%, CHN$_{exp}$: C: 11.00%, H: 1.88%). Yield: 90%, black solid.

Similarly, the other metal ferrite nanoparticles were synthesized by the first microwave-assisted method (MW1) using 1:2 molar ratio of the metal precursors and named MW1_OA@Co$_y$Fe$_{3-y}$O$_4$ and MW1_OA@Mn$_x$Fe$_{3-x}$O$_4$. The cobalt ferrite synthesis yielded about 88% (12.3 mg/3 mL, 61.5 mg total mass, TGA residual mass after N_2 segment: 70.24%, CHN$_{exp}$: C: 15.42%, H: 2.49%, experimental chemical formula as obtained by ICP-MS: $Co_{0.90}Fe_{2.10}O_4$) and the manganese ferrite yielded about 91% (9.5 mg/3 mL, 47.5 mg total mass, TGA residual mass after N_2

segment: 80.69%, CHN_{exp}: C: 11.17%, H: 1.89%, experimental chemical formula as obtained by ICP-MS: $Mn_{0.41}Fe_{2.59}O_4$).

Synthesis of superparamagnetic $Co_yFe_{3-y}O_4$ nanoparticles using microwave-assisted method_ Method 2 (MW2)

$Co_yFe_{3-y}O_4$ nanoparticles were produced using a microwave-assisted method based on a modified literature procedure.[2] For synthesis of MW2_NP1 to MW2_NP5 samples, 0.064 g (0.25 mmol) cobalt (II) acetylacetonate, 0.176 g (0.5 mmol) iron (III) acetylacetonate, 3 mL oleic acid and 7.5 mL oleyl amine were mixed with 1.5 mL benzyl ether in 30 mL microwave vials (G30) and magnetically stirred at room temperature for 10 min to provide homogeneous dark red mixtures. For the synthesis of MW2_NP5_1Diol to MW2_ NP5_5Diol samples, various molar ratios of 1,2-dodecandiol with respect to the molar amounts of precursors (Table S1) were mixed with similar molar amounts of above-mentioned chemicals in G30 vials and stirred at the same conditions to provide homogeneous mixtures. Particles were obtained under optimization of various heating rates and temperatures. Initially, a 10-minute heating at 200 °C facilitated precursor decomposition and nucleation, followed by an additional 10-minute period to complete seed formation. For the growth phase, the reaction mixture was heated to 300 °C within 15 minutes, resulting in rapid pressure increase (up to ~14 bar) from CO and CO_2 production during precursor decomposition (Table S2, MW2_NP1).[2] With the purpose of complete formation of the seeds, which results in higher monodispersity of the particles, a lower heating rate was employed during the nucleation step by extending the duration of the first heating step from 10 to 30 min. Additionally, the effects of different heating rates during the second heating step on the particle formation were investigated (Table S2, MW2_NP2, 3). To reduce the reaction pressure, lower temperature (250 °C) was applied

during the particle growth step (Table S2, MW2_NP4). Furthermore, the impact of heating rate during particle growth step on the uniformity and size of the cobalt ferrite particles was monitored (Table S2, MW2_NP5). Finally, a systematic study was performed using various molar ratios between precursor and 1,2-dodecandiol to investigate the impact of diol on the properties of cobalt ferrites (Table S2, MW2_NP5_1Diol to MW2_NP5_5Diol).

To remove organic residue, about 10 mL of ethanol was added to each of the samples and centrifuged at 7000 rpm for 15 min. The washing step was repeated 3 more times, and finally the oleic acid coated particles were stored under ethanol to avoid oxidation.

Table S1. Experimental molar ratios used in the synthesis of cobalt ferrites with MW2 method.

Sample code	$Co(acac)_2$ (mmol)	$Fe(acac)_3$ (mmol)	1,2-dodecandiol (mmol)
MW2_NP5_1Diol	0.25	0.5	0.75
MW2_NP5_3Diol	0.25	0.5	2.25
MW2_NP5_5Diol	0.25	0.5	3.75

Table S2. Reaction conditions for synthesis of ferrite nanoparticles using MW2 method.

Sample code	1st heating step	2nd heating step	Maximum pressure during reaction (bar)
MW2_NP1	RT to 200 °C in 10 min, holding for 10 min	200 to 300 °C in 15 min, holding for 5 min	14.3-14.7
MW2_NP2	RT to 200 °C in 30 min, holding for 10 min	200 to 300 °C in 15 min, holding for 5 min	15.4-15.8
MW2_NP3	“	200 to 300 °C AFAP*, holding for 5 min	15.0-15.3

MW2_NP4	"	200 to 250 °C AFAP*, holding for 5 min	2.2-2.5
MW2_NP5	"	200 to 250 °C in 30 min, holding for 5 min	3.5-4.0
MW2_NP5_1Diol	"	"	6.4-6.6
MW2_NP5_3Diol	"	"	8.0-8.4
MW2_NP5_5Diol	"	"	6.8-7.1

*AFAP: reaching the goal temperature as fast as possible

FTIR (TD, MW1)

In the FTIR spectra of the nanoparticles, the sharp peak at around 560 cm^{-1} is observed, corresponding to the stretching of metal ions at tetrahedral sites (Figure S1). In pure oleic acid, the strong signal at 1708 cm^{-1}, attributed to the C=O stretching vibration, disappears when oleic acid is bound to the nanoparticle surface. Instead, two distinct signals emerge at 1528 and 1398 cm^{-1} which are characteristic for asymmetric and symmetric stretching vibration of COO^{-}, confirming successful carboxylate bonding of oleic acid to the particles as surfaces. Additionally, two bands at 2917 and 2846 cm^{-1} are associated with the asymmetric and symmetric CH_2 and CH_3 stretching vibrations of the oleic acid chain.[3, 4] Notably, oleic acid has a stronger affinity for binding to metal ions at the surface than oleyl amine.[5] Consequently, no FTIR signals indicative of oleyl amine are detected on the nanoparticle surfaces, suggesting that in systems utilizing both oleic acid and oleyl amine, the oleyl amine primarily facilitates the deprotonation of oleic acid without bonding directly to the particles (Figure S1).[5]

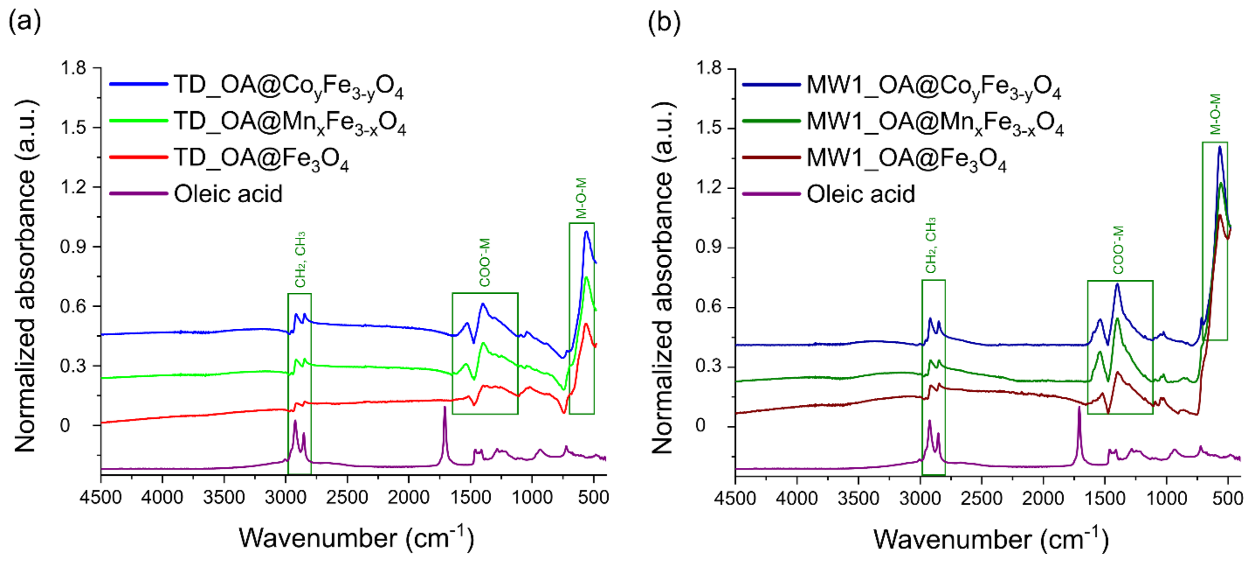

Figure S1. FTIR spectra of oleic acid coated particles synthesized with (a) TD method and (b) MW1 method in comparison with pure oleic acid.

TGA (TD, MW1)

Thermogravimetric analysis (TGA) was conducted to determine the oleic acid content on particle surfaces (Figure S2). Mass loss below 100 °C was consistently below 1% for all samples, indicating minimal residual solvent or adsorbed water. In the samples obtained from both synthesis methods, two distinct steps of mass loss were observed. The first step, from 160 °C to 500 °C under a nitrogen atmosphere, corresponds to the decomposition of oleic acid either physically adsorbed or bonded to the surface. Since in this region only one step of mass loss can be detected, particles were coated with a monolayer of oleic acid.[4] The second mass loss, observed between 500 to 750 °C, is likely due to the partial reduction of particles at higher temperatures, resulting in the formation of metal (II) oxides (CoO, MnO, FeO) along with CO and CO_2 from residual carbon degradation.[6, 7] For particles synthesized via both TD and MW1 methods, mass loss from 500 °C to 750 °C was larger for ferrites than for magnetite, likely due to higher oleic acid coverage

promoting the formation of additional reductive species, thus enhancing reduction in this region. Heating the samples from 900 to 1000 °C under synthetic air resulted in a 3-8% mass gain, caused by the oxidation of M^{2+} species (Figure S2). These findings align with ICP-MS results on M^{2+} substitution levels: manganese-substituted particles exhibited smaller mass increases in this segment compared to magnetite and cobalt ferrite, likely due to lower Mn^{2+} content (Table S3). However, precise quantification of M^{2+} species based on TGA is limited due to the oxidation of the residual carbon of the surface capping agents in the same temperature range.

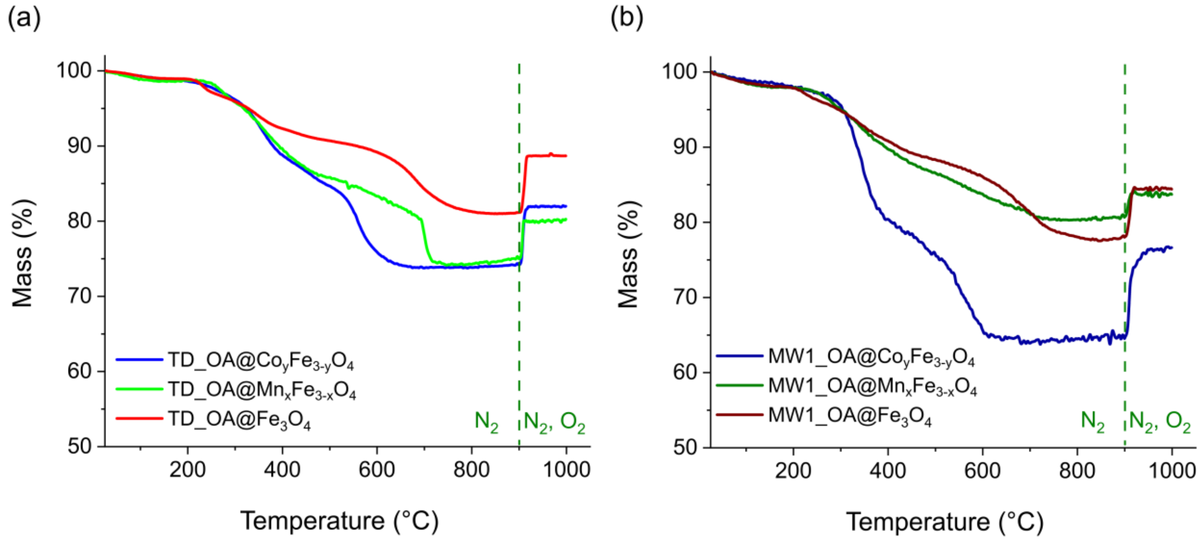

Figure S2. TGA graphs of oleic acid coated particles synthesized with (a) TD method and (b) MW1 method measured under nitrogen and synthetic air atmosphere.

Table S3. TGA mass variations of particles synthesized with TD and MW1 method.

Sample code	Experimental composition (ICP-MS)	mass loss (%) 100-500 °C	mass loss (%) 500-900 °C	Mass increase (%) 900-1000 °C
TD_OA@$Co_yFe_{3-y}O_4$	$Co_{0.85}Fe_{2.15}O_4$	14.68	10.42	7.76
TD_OA@$Mn_xFe_{3-x}O_4$	$Mn_{0.23}Fe_{2.77}O_4$	13.12	10.71	5.22
TD_OA@Fe_3O_4	-	8.00	4.95	3.38
MW1_OA@$Co_yFe_{3-y}O_4$	$Co_{0.90}Fe_{2.10}O_4$	16.74	12.39	7.93
MW1_OA@$Mn_xFe_{3-x}O_4$	$Mn_{0.41}Fe_{2.59}O_4$	11.92	5.84	3.02
MW1_OA@Fe_3O_4	-	10.22	10.29	6.38

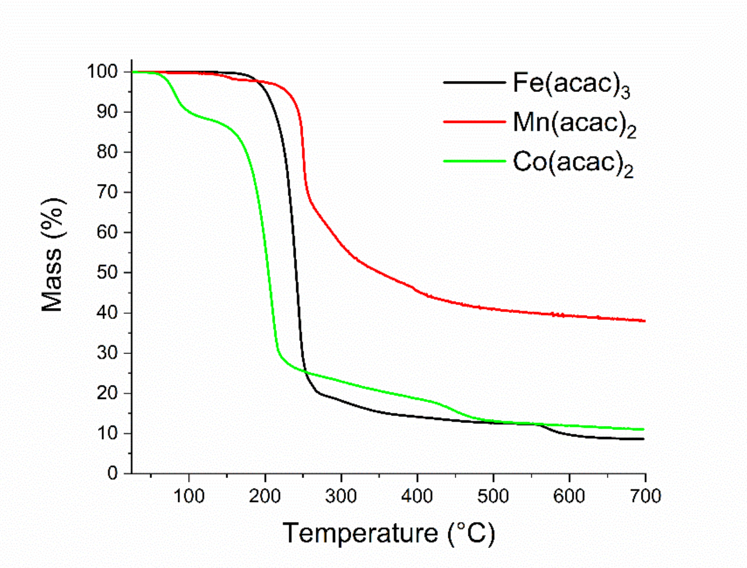

Figure S3. The decomposition temperature of molecular precursors measured under N_2 atmosphere with a heating rate of 10 K/min.

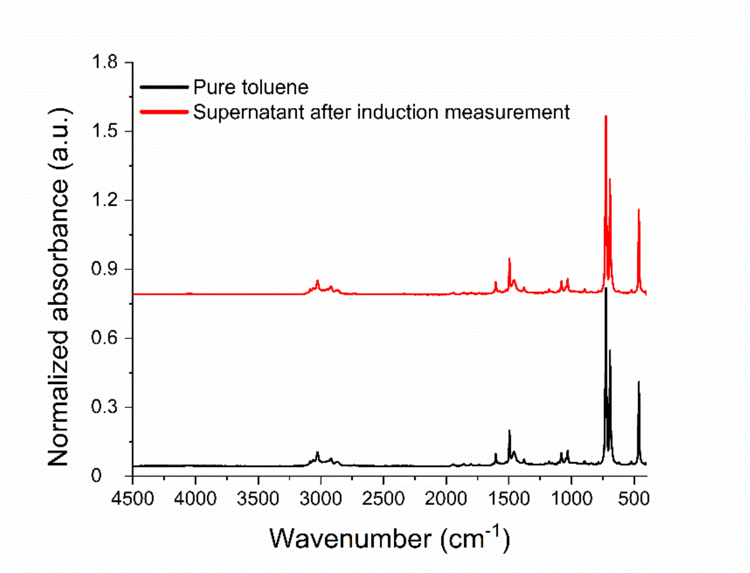

Figure S4. FTIR spectra of MW1_OA@$Co_{0.90}Fe_{2.10}O_4$ particle dispersion supernatant (red) in comparison to pure toluene used as a solvent for induction measurements (black).

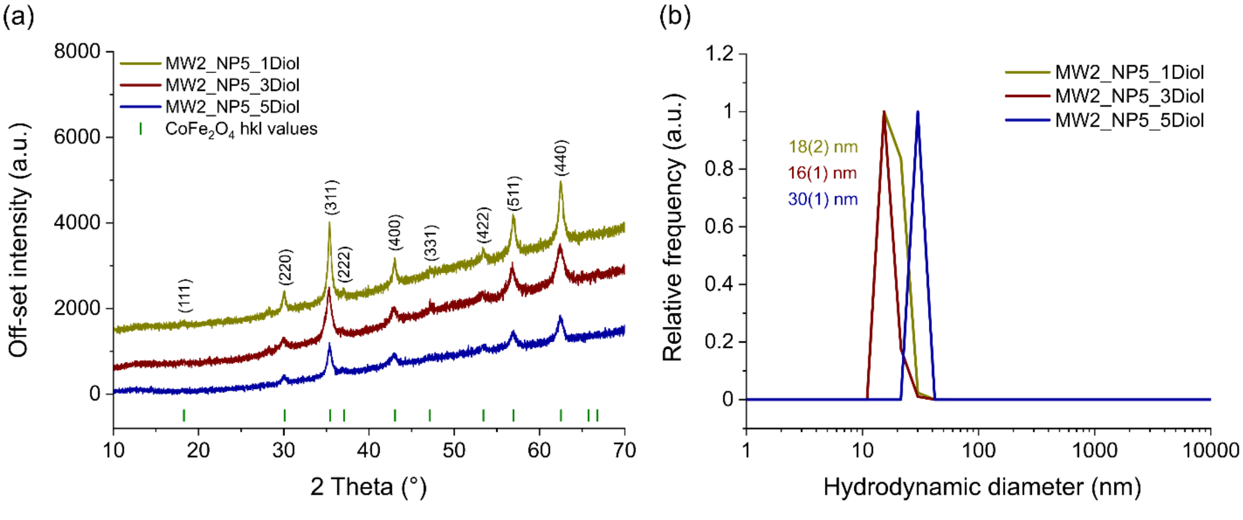

Figure S5. (a) PXRD diffraction patterns of cobalt ferrite nanoparticles synthesized with MW2 method using various amounts of 1,2-dodecandiol in comparison with reference structure[8] and (b) their respective hydrodynamic diameter measured with DLS in n-hexane.

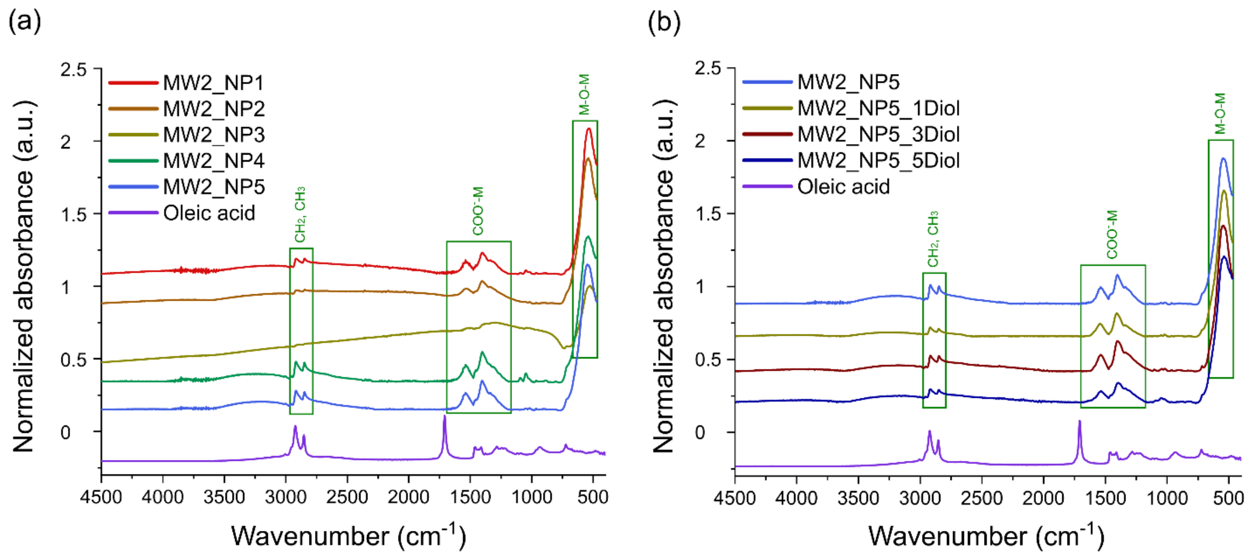

Figure S6. FTIR spectra of cobalt ferrite nanoparticles synthesized with MW2 method in the (a) absence of diol and (b) in the presence of different amounts of 1,2-dodecandiol in comparison with pure oleic acid.

Table S4. Chemical composition of the cobalt ferrite particles synthesized with MW2 and TD method calculated from ICP-MS and EDX analysis data.

**Sample code**	**Chemical composition**	
	**EDX**	**ICP-MS**
MW2_NP1	N. A	$Co_{0.91}Fe_{2.09}O_4$
MW2_NP2	N. A	$Co_{0.90}Fe_{2.10}O_4$
MW2_NP3	N. A	$Co_{0.94}Fe_{2.06}O_4$
MW2_NP4	N. A	$Co_{0.74}Fe_{2.26}O_4$
MW2_NP5	$Co_{0.89}Fe_{2.11}O_4$	$Co_{0.80}Fe_{2.20}O_4$
MW2_NP5_1Diol	$Co_{0.90}Fe_{2.10}O_4$	$Co_{0.90}Fe_{2.10}O_4$
MW2_NP5_3Diol	$Co_{0.90}Fe_{2.10}O_4$	$Co_{0.88}Fe_{2.12}O_4$
MW2_NP5_5Diol	$Co_{0.87}Fe_{2.13}O_4$	$Co_{0.85}Fe_{2.15}O_4$
TD_OA@$Co_{0.85}Fe_{2.15}O_4$	$Co_{0.82}Fe_{2.18}O_4$	$Co_{0.85}Fe_{2.15}O_4$

Impacts of dispersion medium and particle concentration on the heating efficiency of microwave-assisted synthesized cobalt ferrites

Particles exhibited high dispersibility and heating efficiency in low-viscosity Poly(dimethyl siloxane) (PDMS), suggesting similar behavior in non-polar polymers. In higher-viscosity PDMS (100 c.St), particles dispersibility decreased, resulting in lower heating efficiency. Despite the hindrance of particle rotation by the medium, the particles still demonstrated a significant temperature variation (ΔT = 64 °C) over 5 minutes, indicating that Néel relaxation is the main mechanism responsible for heating (Figure S7a). Similar to concentrations higher than 5 mg/mL, at lower concentrations, particles show an increase of temperature by increase of the particle concentration (Figure S7b).

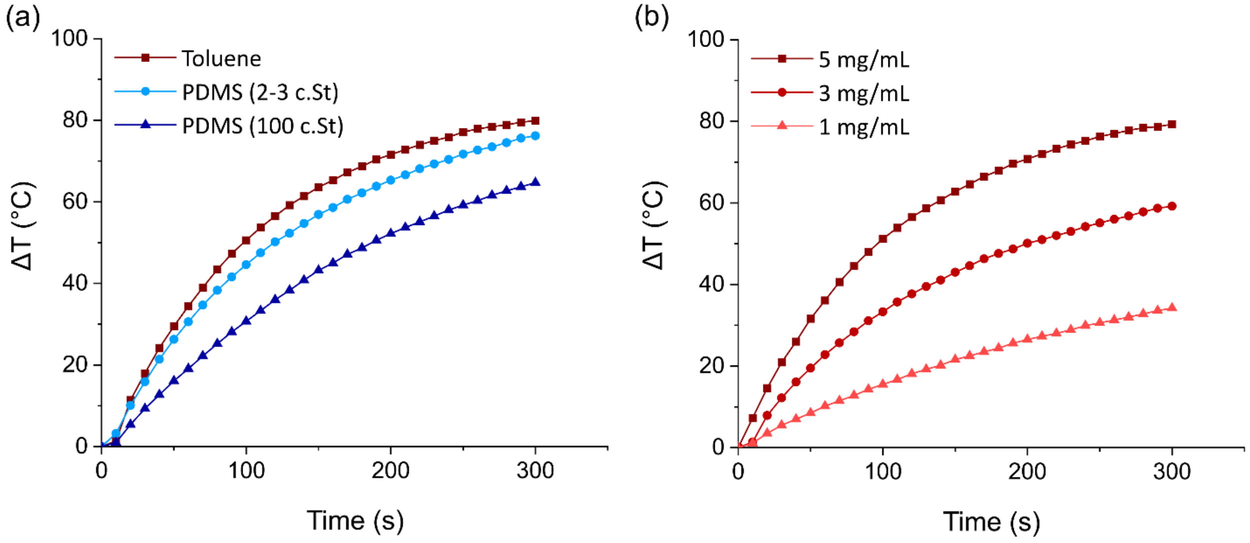

Figure S7. Heating efficiency of MW-assisted synthesized cobalt ferrite particles: (a) in dispersion media with various viscosities and (b) at low particle concentrations.

Table S5. Surface to volume ratio of cobalt ferrite particles with optimized properties calculated from average particle diameter (D_{TEM})

Sample code	Average D_{TEM} (nm)	Nanoparticle volume (nm^3)	Average surface area (nm^2)	Surface/volume ratio
TD_OA@$Co_{0.85}Fe_{2.15}O_4$	7.9	258	196	0.76
MW1_OA@$Co_{0.90}Fe_{2.10}O_4$	5.8	102	106	1.03
MW2_NP5	7.9	258	196	0.76
MW2_NP5_3Diol	8.1	278	206	0.74

Reference

1. S. Sun, H. Zeng, D. B. Robinson, S. Raoux, P. M. Rice, S. X. Wang and G. Li, *J. Am. Chem. Soc.*, 2004, **126**, 273-279.
2. Y.-J. Liang, Y. Zhang, Z. Guo, J. Xie, T. Bai, J. Zou and N. Gu, *Chem. Eur. J.*, 2016, **22**, 11807-11815.
3. L. Zhang, R. He and H.-C. Gu, *Appl. Surf. Sci.*, 2006, **253**, 2611-2617.
4. Y. Urian, J. Atoche-Medrano, L. T. Quispe, L. L. Félix and J. Coaquira, *J. Magn. Magn. Mater.*, 2021, **525**, 167686.
5. R. A. Harris, P. M. Shumbula and H. t. van der Walt, *Langmuir*, 2015, **31**, 3934-3943.
6. K. Yang, H. Peng, Y. Wen and N. Li, *Appl. Surf. Sci.*, 2010, **256**, 3093-3097.
7. C. W. Lai, F. W. Low, M. F. Tai and S. B. Abdul Hamid, *Adv. Polym. Tech.*, 2018, **37**, 1712-1721.
8. N. Modaresi, R. Afzalzadeh, B. Aslibeiki, P. Kameli, A. Ghotbi Varzaneh, I. Orue and V. A. Chernenko, *J. Magn. Magn. Mater.*, 2019, **482**, 206-218.
